# Phylogenetic and Chemotaxonomic Studies Confirm the Affinities of *Stromatoneurospora phoenix* to the Coprophilous Xylariaceae

**DOI:** 10.3390/jof6030144

**Published:** 2020-08-23

**Authors:** Kevin Becker, Sarunyou Wongkanoun, Anna-Charleen Wessel, Gerald F. Bills, Marc Stadler, J. Jennifer Luangsa-ard

**Affiliations:** 1 Department of Microbial Drugs, Helmholtz Centre for Infection Research GmbH (HZI), Inhoffenstraße 7, 38124 Braunschweig, Germany; kevin.becker@helmholtz-hzi.de (K.B.); a.wessel@tu-bs.de (A.-C.W.); 2 German Centre for Infection Research Association (DZIF), Partner Site Hannover-Braunschweig, Inhoffenstraße 7, 38124 Braunschweig, Germany; 3 Faculty of Biotechnology, College of Agricultural Innovation, Biotechnology and Food, Rangsit University, Phahonyothin Road, Lak-Hok, Pathum Thani 12000, Thailand; sarunyou.wong@gmail.com; 4 National Biobank of Thailand (NBT), National Science and Technology Development Agency (NSTDA), 111 Thailand Science Park, Phahonyothin Road, Khlong Nueng, Khlong Luang, Pathum Thani 12120, Thailand; 5 Texas Therapeutics Institute, The Brown Foundation Institute of Molecular Medicine, The University of Texas Health Science Center at Houston, 1881 East Road, Houston, TX 77054, USA; billsge@vt.edu; 6 Plant Microbe Interaction Research Team (APMT), Integrative Crop Biotechnology and Management Research Group, National Center for Genetic Engineering and Biotechnology (BIOTEC), 113 Thailand Science Park, Phahonyothin Road, Khlong Nueng, Khlong Luang, Pathum Thani 12120, Thailand

**Keywords:** secondary metabolites, sesquiterpenoids, Sordariomycetes, structure elucidation, Xylariales

## Abstract

The genus *Stromatoneurospora* was erected in 1973 by Jong and Davis to accommodate the pyrophilic pyrenomycete *Sphaeria phoenix* and has traditionally been placed in the family Xylariaceae based on morphological features. However, no living culture of this genus has so far been available in the public domain. Molecular data were restricted to an internal transcribed spacer (ITS) sequence that only confirmed the familial position, and was generated from a strain that is not deposited in a public culture collection. We have recently collected fresh material and were able to culture this fungus from Thailand. The secondary metabolites of this strains were analysed after fermentation in multiple media. The the prominent components of these fermentation were purified, using preparative chromatography. Aside from two new eremophilane sesquiterpenoids named phoenixilanes A–B (**1**–**2**), four other components that are known from species of the xylariaceous genera *Xylaria* and *Poronia* were identified by spectral methods (nuclear magnetic resonance spectroscopy and high resolution mass spectrometry). Notably, (−)-(*R*)-6-hydroxy-3-methyl-4-dihydroisocoumarin-5-carboxylic acid (**6**) has not been reported as a natural product before. Moreover, DNA sequences of *Stromatoneurospora phoenix* clustered with members of the genera *Poronia* and *Podosordaria* in a multi-locus molecular phylogeny. These results confirmed that the genus belongs to the same evolutionary lineage as the coprophilic Xylariaceae. The results also suggest that this lineage has evolved independently from the plant-inhabiting saprotrophs and endophytes that are closely related to the genus *Xylaria*. These findings are discussed in relation to some theories about the endophytic vs. the pyrophilic/coprophilic fungal life style.

## 1. Introduction

The genus *Stromatoneurospora* was erected in 1973 to accommodate *Sphaeria phoenix* [1]. This pyrenomycete was originally collected by the German botanist Weigelt, and Kunze (in Fries [2]) provided the first description. The epithet “*phoenix*” actually refers to the legendary bird in Greek mythology that burns but never dies and always and again arises from its own ash. Indeed, the holotype of this fungus originates from Suriname, where it was found on “semi-burnt” grasses. The taxonomic affinities of this fungus remained unsettled for a long time, because its light colored stromata are reminiscent of the Hypocreales, while the micromorphology of asci and ascospores point towards affinities to certain taxa in the Xylariales and Sordariales, respectively. It was alternatively assigned to the genera *Xylaria*, *Hypoxylon*, and *Sarcoxylon*. The salient features on which Jong and Davis based their concept for a new genus were the brown, ornamented ascospores that are devoid of germ slits (reminiscent of the genus *Neurospora*) in combination with the presence of stromata. The stromata are lacking the melanization found in the majority of the stromatic Xylariales and are, in this respect, more reminiscent of a hypocrealean taxon (referring to the classical definition based on macromorphology). However, the crucial fact that led Jong and Davis to finally assign their new genus to the Xylariaceae was the ascal morphology, which is typical of the Xylariales. They described in detail the stipitate cylindrical asci with an amyloid apical apparatus as follows: “In face view the ring is doughnut-shaped. In optical cross section it is shaped like an inverted hat, i.e., wider at the top than at the bottom [1].” Modern taxonomic studies involving multi-locus genealogies have proven in retrospective that this character is more predictive of the phylogenetic affinities than the shape of the ascospores. This was the reason why *Stromatoneurospora* has been retained in the Xylariaceae s. str., even after the recent segregation of families in the stromatic Xylariales, which were based on a combination of molecular phylogenetic data, holomorphic morphology, and (to a great extent) even chemotaxonomic evidence [3,4].

However, no data on the anamorphic structures have so far been published, and the molecular data on *Stromatoneurospora* in the public domain were scarce and unreliable. An internal transcribed spacer (ITS) sequence labeled “*S. phoenix*” has been deposited in GenBank (Acc. No. AY909004) and the entry refers to an early study of the phylogeny of the Xylariales [5]. However, this sequence was not employed in the phylogenetic analyses reported in the paper cited in the GenBank entry, which, for now, also incorrectly gives the country of origin as “USA”. Actually, our inquiries with V. Gonzalez (Fundacion MEDINA, pers. comm.) have revealed information that the DNA sequence deposited in GenBank is derived from a specimen identified as *Stromatoneurospora phoenix* by one of the authors (G.F.B.). The culture (designation GB 6330) was originally isolated from a soil sample collected in Mexico, Veracruz, La Joya, in December of 1999 after ethanol treatment, and formed the typical stromata of *S. phoenix* on various media. Today it is preserved at the collection of Fundacion MEDINA (Granada, Spain).

Another sequence labeled “*Stromatoneurospora* sp.” with the Acc. No. MH430290 was published as an Operational Taxonomic Unit (OTU) in a study of the mycobiome of *Vitis vinifera* [6], but the DNA was only detected by methods of next generation sequencing (NGS). No culture resulted from the latter study. BLAST searches and alignments of both sequences suggested that the corresponding fungi belong to the Xylariaceae, but there was no hard evidence on their phylogenetic position. As recently demonstrated based on high quality genome data, ITS sequences are not well suited for discrimination of genera and species in the Xylariales because of intragenomic polymorphisms [7], and have been shown, repeatedly, not to resolve genera in this order [3,4]. Multi-locus genealogies are therefore called for when it comes to the phylogenetic placement of hitherto unstudied fungi in the Xylariales.

We have recently encountered a specimen in Thailand whose morphological features matched the descriptions of *Stromatoneurospora* and were able to obtain a mycelial culture from its ascospores. The present paper is dedicated to report its phylogenetic affinities as inferred from a multi-locus phylogeny, supported by the investigation of its secondary metabolites.

## 2. Materials and Methods

### 2.1. Survey and Sample Collection

Stromatic Xylariales were collected in community forests and national parks in Thailand. Photographs were taken using 60D digital camera (Canon, Tokyo, Japan). Fungal cultures were obtained using the multiple spore isolation method as described in Sir et al. [8]. Germinated ascospores were transferred to new agar plates. Axenic cultures and voucher specimens were deposited in the BIOTEC Culture Collection (BCC) and BIOTEC Bangkok Herbarium (BBH), Thailand, respectively. Scanning Electron Microscopy (SEM) was carried out using a conventional procedure as described previously [9].

### 2.2. Morphological Characterization

Morphological characters, such as stromatal sizes and shapes, perithecia, asci and ascospores were examined following Wendt et al. [4] using a compound microscope Olympus ZX31 (Olympus, Tokyo, Japan) and a stereo microscope Olympus SZ61 (Olympus). Fungal cultures were grown on oatmeal agar (Difco: OA) for morphological studies. Conidiogenous cells and conidiophore branching patterns of the anamorph were studied according to [3]. Furthermore, the color of fresh stromata and cultures were recorded using the color chart of Rayner [10] and the capitalized color codes proposed in this chart are given.

### 2.3. DNA Extraction, Polymerase Chain Reaction (PCR), and Sequencing

A method based on cetyltrimethyl ammonium bromide (CTAB) was used to extract total genomic DNA from the mycelia according to [11]. The internal transcribed spacer (ITS) regions, and large subunit (LSU) of the rDNA, partial regions of the RNA polymerase II (*RPB2*), and *β*-tubulin (*TUB2*) genes were amplified, following the standard primers introduced by White et al. ([12]; ITS4 and ITS5 for ITS rDNA), Vilgalys and Hester ([13]; LR7 and Bunyard et al. ([14]; LROR) for LSU), Liu et al. ([15]; *RPB2*–5F and 7Cr for *RPB2*), and O’Donnell and Cigelnik ([16]; T1 and T22 for *TUB2*), according to a previously published protocols [4]. The PCR products were sent to Macrogen Co. (Seoul, Korea) for purification and sequencing using the same primers used for the PCR amplification reaction. DNA sequences were checked and assembled using BioEdit v. 7.2 [17]. All newly generated sequences were submitted to GenBank (https://www.ncbi.nlm.nih.gov/) and listed in Table 1.

### 2.4. Phylogenetic Analyses

All sequences were aligned in Multiple Sequence Comparison by Log-Expectation (MUSCLE) [18] and refined by direct examination. Multiple sequence alignments were analyzed with closely matching sequences and other reference taxa obtained from GenBank, as shown in Table 1. Sequences were analyzed using maximum parsimony (MP), maximum likelihood (ML), and Bayesian algorithm (MB). The MP analysis was performed in PAUP*4.0b10 (https://paup.phylosolutions.com/); all characters were equally weighted and gaps were treated as missing data. The most parsimonious trees were obtained from the heuristic searches: 100 replicates of random stepwise addition of sequence, branch-swapping algorithm: tree-bisection-reconnection (TBR) and equal weight characters. Maximum parsimony bootstrap values were estimated by 1000 replicates (stepwise addition of sequence, 10 replicates of random addition of taxa, TBR branching-swapping algorithm). Tree length (TL), consistency index (CI), retention index (RI), relative consistency index (RC) and homoplasy index (HI) were estimated. The ML tree and bootstrap analyses were conducted through the CIPRES Science Gateway V 3.3 [19] using RAxML 8.2.4 [20] with the Broyden Fletcher Goldfarb Shanno (BFGS) method to optimize General Time Reversible (GTR) rate parameters. Bayesian posterior probabilities of the branches were performed using MrBayes 3.0B4 [21] with the best-fit model (GTR + I + G) selected by Akaike Information Criterion (AIC) in MrModeltest 2.2 [22], tested with hierarchical likelihood ratios (hLRTs). Three million generations were run in four Markov chains and sampled every 100 generations with a burn-in value set at 3000 sampled trees. Sequences of *Daldinia concentrica* and *Hypoxylon fragiforme* (Hypoxylaceae) were used as outgroups. For comparison of the DNA sequences from GenBank, a separate tree was constructed based on ITS data, aside from the multi locus tree. The phylogenetic trees revealed by RAxML are depicted as phylograms in Figures 3 and 4.

### 2.5. Analytical Equipment for Structure Elucidation

Electrospray Mass (ESI-MS) spectra were recorded with an UltiMate^®^ 3000 Series UHPLC system (Thermo Fisher Scientific, Waltman, MA, USA) connected to an amaZon speed^®^ ESI-Ion Trap-MS (Bruker, Billerica, MA, USA) mass spectrometer, utilizing a C18 Acquity^®^ UPLC BEH column (2.1 × 50 mm, 1.7 µm; Waters, Milford, MA, USA) as stationary phase. HPLC parameters were set as follows: solvent A: H_2_O + 0.1% formic acid, solvent B: acetonitrile (ACN) + 0.1% formic acid; gradient 5% B for 0.5 min, increasing to 100% B in 19.5 min, keeping 100% B for further 5 min; flow rate 0.6 mL/min, with diode array (DAD) detection in the range of 200–600 nm.

High Resolution Electrospray Mass (HR-ESI-MS) spectra were obtained with an Agilent 1200 Infinity Series HPLC (Agilent Technologies, Santa Clara, CA, USA) connected to a maXis^®^ Electrospray Time-of-flight mass spectrometer (ESI-TOF-MS; Bruker). The HPLC conditions were the same as for ESI-MS measurements.

NMR spectra were recorded with an Avance III 500 spectrometer (Bruker, ^1^H NMR: 500 MHz, ^13^C NMR: 125 MHz). Optical rotations were taken with a MCP 100 circular polarimeter (Anton Paar, Graz, Austria) and UV/vis spectra with a UV-2450 spectrophotometer (Shimadzu, Kyoto, Japan). ECD spectra were recorded on a J-815 spectropolarimeter (JASCO, Pfungstadt, Germany).

### 2.6. Fungal Material and Cultivation

All DNA sequences of representative reference specimens are listed in Table 1. The taxonomy of fungal names follows MycoBank (http://www.mycobank.org/, accessed on 5 July 2020) and therefore the authorities are not given here.

Stromata of *Stromatoneurospora phoenix* were collected in Ban Hua Thung community forest, Chiang Mai Province, Thailand, on burnt grass on 6 July 2016, by P. Srikitikulchai and S. Wongkanoun. The specimens are deposited under the designation number BBH42282 at the BIOTEC herbarium (Pathum Thani, Thailand), and the corresponding cultures, which were obtained from multiple ascospores in the BIOTEC culture collection (dto.) under the designation numbers BCC82040 and BCC82041. Both strains, BCC82040 and BCC82041 were used for the phylogenetic analysis and only BCC82040 was used in the chemical analysis.

The following strains of coprophilic Xylariaceae (all isolated by G.B. from dung incubated in a most chamber) were used for comparison in the molecular phylogeny, and have been deposited at the DSMZ (Braunschweig, Germany):

*Hypocopra anomala;* TTI-000339, USA, Rt. 90, Amistad Village, Val Verde Co., TX, rabbit dung;

*Hypocopra dolichopoda;* TTI-000310, USA Rt. 90, near Brackettville, Kinney Co., TX, rabbit dung;

*Hypocopra rostrata;* TTI-000009, USA, Jack Brooks Park, Hitchcock, Galveston Co., TX, horse dung;

*Podosordaria leporina;* TTI-000312, USA, Rt. 90, near Brackettville, Kinney Co., Texas, rabbit dung.

For fermentation of *S. phoenix,* the seed culture was conducted in 250 mL flasks each containing 50 mL semi-viscous SMYA medium [35] (maltose 40 g/L, yeast extract 10 g/L, meat peptone 10 g/L, agar 4 g/L). Inoculation was done by adding five pieces (ca. 25 mm^2^ each) of a well-grown agar-plate of *S. phoenix* to each vessel. These flasks were inoculated for 3 d on a shaker (23 °C, 140 rpm).

Different media were utilized for the production cultures. Each flask, regardless of the medium used, was inoculated with 3 mL of the seed culture and incubated at 23 °C for 14 d. Submerged cultures were shaken at 140 rpm during the time of cultivation. Two liquid media were used for submerged production cultures. Each flask (500 mL) was filled with 200 mL of ZM ½ medium (21 flasks; molasses 5 g/L, oatmeal 5 g/L, sucrose 4 g/L, mannitol 4 g/L, d-glucose 1.5 g/L, CaCO_3_ 1.5 g/L, edamin 0.5 g/L, (NH_4_)_2_SO_4_ 0.5 g/L; pH 7.2), and YM 6.3 medium (20 flasks; malt extract 10 g/L, d-glucose 4 g/L, yeast extract 4 g/L; pH 6.3), respectively [31].

The fungus was also cultivated in solid state using BRFT medium [36] (brown rice 25 g as well as 0.1 L of base liquid (yeast extract 1 g/L, sodium tartrate 0.5 g/L, KH_2_PO_4_ 0.5 g/L) per flask. First, the rice was weighed into flasks and the base liquid added, followed by autoclaving).

### 2.7. Extraction and Isolation of Secondary Metabolites

The extraction procedures of cultures to gain the respective crude extracts are described below. However, only the isolation of the two novel secondary metabolites **1** and **2** is described herein, while the respective steps are described in the Supplementary Information for the known compounds **3**−**6**.

Production cultures were harvested 14 d after inoculation. For submerged cultures (ZM ½, YM 6.3), the supernatant and the mycelium were separated by filtration through gauze. The aqueous supernatants (*ca.* 4 L each) were extracted twice with a separatory funnel using equal amounts of ethyl acetate (EtOAc), respectively. Both extracts were combined and dried in vacuo at 40 °C to yield an oily crude extract, respectively.

The solid cultures (BRFT) were pooled in one glass bottle, covered with acetone, and sonicated in an ultrasonic bath (1 h, 40 °C). Gauze was used to separate the acetone from the mycelium, and the latter was again subjected to the sonication procedure. Both acetone extracts were combined and dried in vacuo at 40 °C. Then, the remaining solid was dispersed in 1 L of H_2_O and extracted twice, using 1 L of EtOAc. The extracts were combined, dried in vacuo at 40 °C, and weighed. Crude extract yields were as follows: ZM ½: 807 mg, YM 6.3: 775 mg, and BRFT: 800 mg.

For isolation of **1** and **2**, the crude extract from ZM ½ medium was portioned to 3 × 270 mg and separated using a PLC 2250 preparative HPLC system (Gilson, Middleton, WI, USA) with a Nucleodur^®^ C18ec column (125 × 40 mm, 7 µm; SN 762042.400, Macherey-Nagel, Düren, Germany) as stationary phase, and the following conditions: solvent A: H_2_O + 0.1% formic acid, solvent B: ACN + 0.1% formic acid; flow: 50 mL/min, fractionation: 20 mL, gradient: isocratic conditions at 5% B for 5 min, followed by an increase to 55% B in 50 min, then increase to 100% B in 5 min, followed by isocratic conditions of 100% B for 10 min. This yielded the pure fraction #78–82 of **1**, 101 mg, *t*_R_ = 42–45 min) as well as the yet impure #60 (20.5 mg, *t*_R_ = 32–33 min).

Fraction #60 was further separated via preparative thin layer chromatography (TLC) using SILGUR UV254 glass plates (200 × 200 mm, 0.25 mm silica layer thickness; SN 810023, Macherey-Nagel). As eluent, 150 mL of dichloromethane (DCM): acetone 1:1 was used. This yielded 3.8 mg of **2** (*R*_f_ = 0.59–0.70).

### 2.8. Antimicrobial and Cytotoxic Activity Assay

Compounds **1**, **2**, and **4** were dissolved in MeOH (1 mg/mL) for the bioactivity assays. The solvent was also used as negative control.

Minimum inhibitory concentrations (MIC) were determined in a serial dilution assay to assess the antimicrobial effects of the test compounds as described previously [37]. Various test organisms of fungal and bacterial origin were tested to cover a broad range of microorganisms. Bacteria: *Bacillus subtilis*, *Staphylococcus aureus*, *Micrococcus luteus*, *Chromobacterium violaceum*, *Escherichia coli*, and *Pseudomonas aeruginosa*; mycobacteria: *Mycolicibacterium smegmatis* and fungi: *Candida albicans*, *Schizosaccharomyces pombe*, *Mucor hiemalis*, *Pichia anomala,* and *Rhodotorula glutinis*.

The cytotoxicity was evaluated in assays against the cell lines L929 (mouse fibroblasts) and KB 3.1 (human papillomavirus-related endocervical adenocarcinoma) as described previously [38]. If inhibition of cell viability with an IC_50_ < 50 µM was observed, further cell lines were subjected to the respective compounds: PC-3 (human prostate adenocarcinoma), SK-OV-3 (human ovary adenocarcinoma), MCF-7 (human breast adenocarcinoma), A431 (human squamous carcinoma), and A549 (human lung carcinoma).

### 2.9. Spectral Data

#### 2.9.1. Phoenixilane A (**1**)

Colorless solid. [*α*]_D_ = −77 (*c* 0.1, MeOH); NMR (acetone-*d_6_*, ^1^H NMR: 500 MHz, ^13^C NMR: 125 MHz), see Table 2; UV/vis (c = 0.01 mg/mL, ACN): *λ*_max_ (*ε*) = 245 (3.7) nm, see Figure S3; ECD (c = 1 mg/mL, ACN): *λ*(*Δε*): 218 (+10.5), 248 (−8.4), 281 (−0.2), 331 (−2.3) nm, see Figure S4; ESI-MS: *m/z* 249.05 [M + H]^+^, 247.03 [M − H]^−^; HR-ESI-MS: *m/z* 249.1483 [M + H]^+^ (calculated for C_15_H_21_O_3_, 249.1485); *t*_R_ = 7.9 min. NMR spectra see Figures S5–S12.

#### 2.9.2. Phoenixilane B (**2**) 

Colorless solid. [*α*]_D_ = −54 (*c* 0.1, MeOH); NMR (DMSO-*d_6_*, ^1^H NMR: 500 MHz, ^13^C NMR: 125 MHz), see Table 2; UV/vis (c = 0.01 mg/mL, MeOH): *λ*_max_ (*ε*) = 244 (3.8) nm, see Figure S3; ECD (c = 1 mg/mL, MeOH): *λ*(*Δε*): 197 (+7.2), 215 (−3.4), 221 (+3.5), 251 (−3.5), 285 (−0.2), 330 (−2.0) nm, see Figure S4; ESI-MS: *m/z* 265.09 [M + H]^+^, 263.00 [M − H]^−^; HR-ESI-MS: *m/z* 287.1254 [M + Na]^+^ (calculated for C_15_H_20_O_4_Na, 287.1254); *t*_R_ = 7.2 min. NMR spectra see Figures S13–S18.

## 3. Results

### 3.1. Taxonomic and Phylogenetic Characterization

The morphological characteristics of the two specimens of *Stromatoneurospora phoenix* and the phylogenetic position of this taxon according to a multi-locus genealogy are described further below.

*Stromatoneurospora phoenix* (Kunze ex Fr.) S.C. Jong and E.E. Davis, Mycologia 65: 459 (1973), Figure 1.

*Materials studied*: Thailand, Chiang Mai Province, Ban Hua Thung community forest, 19.42044′ N, 98.97140′ E, on burnt grass, 6 July 2016, P. Srikitikulchai, S. Wongkanoun, BBH 42282, corresponding cultures BCC82040 and BCC82041, independently obtained from two different stromata of BBH-42282); GenBank accession numbers for DNA sequences are presented in Table 1.

*Teleomorph*. *Stromata* scattered on the host surface, subglobose to ovate, stipitate, roughened, 2–6 mm diameter, stipes short, slender, unbranched, smooth, black, 2–2.2 mm long, deeply rooting in the substrate; externally Tawny Blended (8), Umber (9) or Apricot (42) with black papillate ostioles of embedded perithecia, internally white. Texture fairly hard, lacking carbonaceous layer. *Perithecia* completely immersed beneath the stroma, surface obovoid to globose, 650–850 × 750–100 μm; ostioles conspicuous, black, papillate. Paraphyses typical of the stromatic Xylariales, tapering, 3–5 septate, (200–) 300–325 × 5–7.5 μm (M = 283.69 × 5.97 µm; n = 50). *Asci* unitunicate, eight-spored, cylindrical, (150) 175–200 × (7.5–) 10–12.5 μm. *Ascospores* ellipsoid-fusiform, 1-celled, hyaline at first, becoming yellow brown and black in maturity, (15–)18–20(–21) × (7–)8–9(–10) μm, (M = 18.7 × 8 µm; n = 50) with longitudinal, parallel to convergent, continuous to discontinuous ridges on the wall resembling those of *Neurospora* ascospores and have neither germ pore nor germ slits.

*Anamorph in culture* lindquistia-like. *Synnemata* cylindrical to clavate, 24–25 × 2–3 mm. *Conidiophores* loosely arranged, branched, undetermined in length, 2–3 μm diameter. *Conidiogenous cells* produced holoblastically, cymbiform, rarely subglobose to obovoid, hyaline, 9–10 × 4–5 μm, each cell producing one or several conidia. *Conidia* hyaline, smooth, subglobose, obovoid, ellipsoid with flattened base, 4–6 × 2–3 μm.

**Figure 1:**
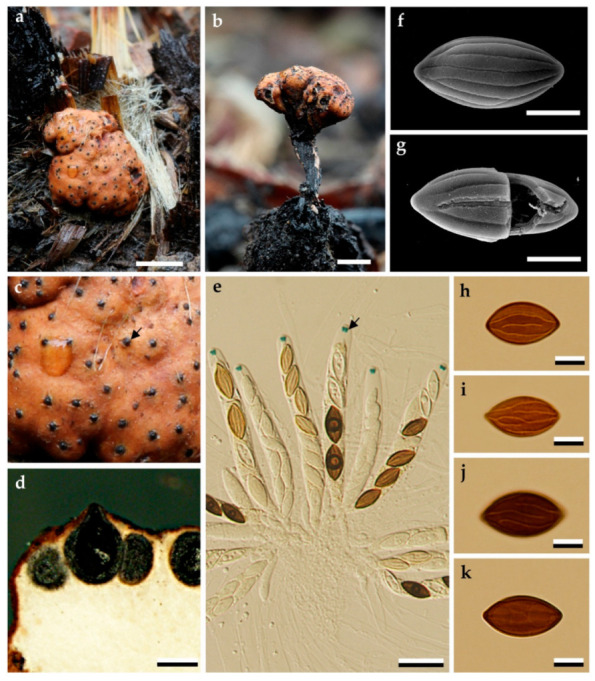
Morphological characteristics of *Stromatoneurospora phoenix* (specimen BBH 42282). (**a**,**b**): stromata in the natural habitat; (**c**): stromatal surface and ostioles; (**d**): longitudinal section of stroma showing perithecia and the tissue below the perithecial layer; (**e**): asci with apical apparatus bluing in Melzer’s reagent (black arrow); (**f**,**g**): ascospores by scanning electron microscopy (SEM); (**h**–**k**): ascospores by light microscopy. Scale is indicated by bars ((**a**): 2 mm. (**b**): 1 mm. (**d**): 500 µm; (**e**): 20 µm, (**f**–**k**): 5 µm).

**Figure 2:**
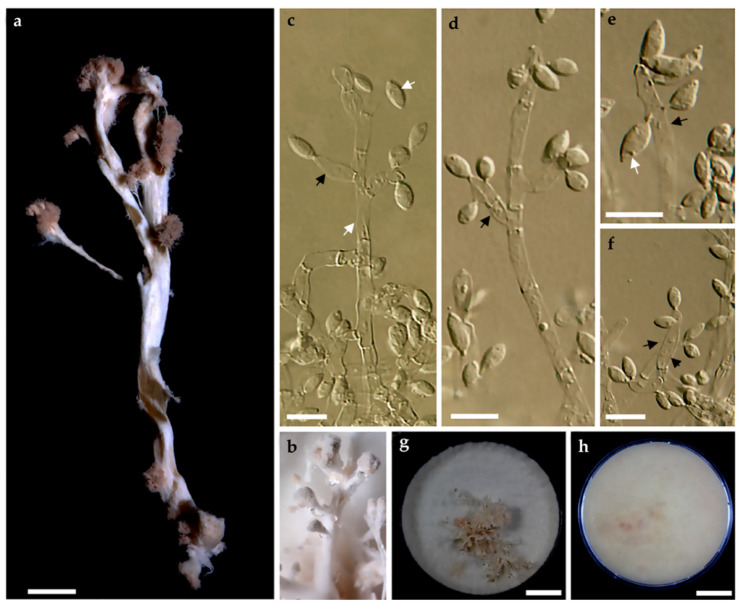
Culture characteristics and lindquistia–like anamorph of *Stromatoneurospora phoenix* strain BCC82040; (**a**): mature synnema in culture; (**b**): young synnema in culture; (**c**–**f**): conidiogenous cells (indicated by black arrows) and conidia; conidiophore indicated in d by white arrow; (**g**,**h**): colony on OA after one month. Scale is indicated by bars: (**a**) scale bar = 2 mm; (**c**–**f**) scale bar = 10 µm; (**g**,**h**) scale bar = 2 cm).

*Culture characteristics.* Colonies on OA reaching the edge of a 9 cm Petri dish in 1 week, at first whitish becoming velvety to felty, azonate with entire margin, peach (4), flesh (37), or salmon (41) after 1 month incubation (Figure 1g,h). Synnemata produced after 1 month of incubation at room temperature (*ca.* 20–25 °C; Figure 2a,b). Colonies on YMGA covering Petri dish in a week, at first whitish, becoming peach (4), flesh (37), and salmon (41), velvety to felty, azonate with entire margin.

*Notes*. The morphological characteristics of our fungus are clearly similar to those of the holotype of *Stromatoneurospora phoenix* that was reported from Surinam, as well as to specimens that were later reported from Brazil, Puerto Rico, and USA. Aside from *S. phoenix* there is only one other species that was assigned to the genus, i.e., *Stromatoneurospora elegantissima,* reported from burnt grass in Brazil. In keeping with the description of Jong and Davis [2], this species differs from *S. phoenix* in having much larger ascospores (25 × 12 μm). Three other genera are morphologically similar to *Stromatoneurospora* by having a fairly hard stromatal texture, lacking a carbonaceous layer, and some of them also produce a lindquistia-like anamorph: *Podosordaria*, *Poronia,* and *Sarcoxylon* also show affinities with *Stromatoneurospora* but differ in their ascospore morphology.

### 3.2. Molecular Phylogeny

**Figure 3:**
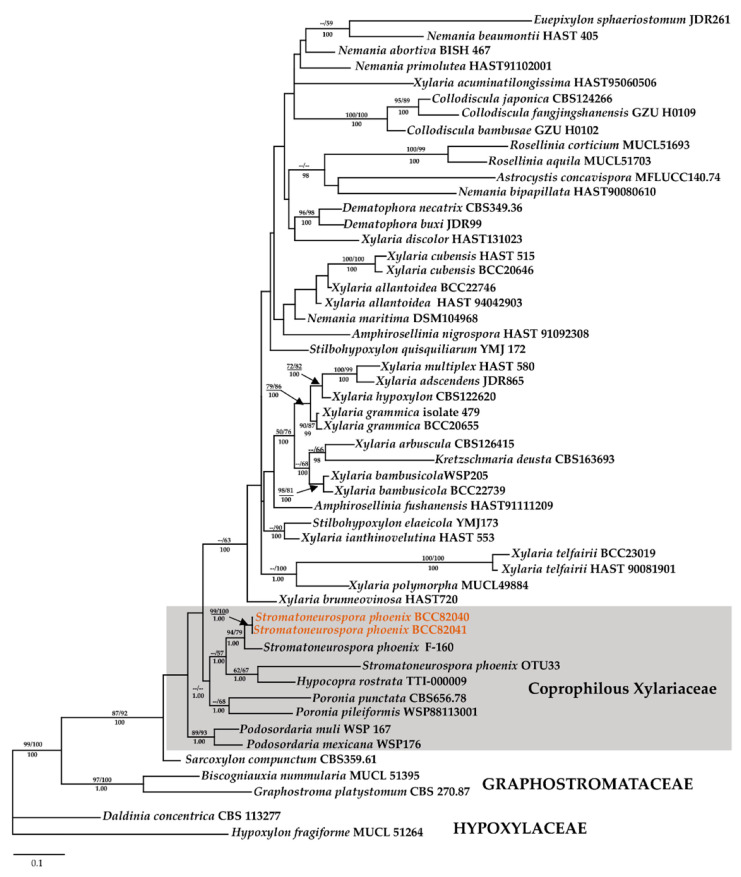
Phylogenetic relationships inferred from RAxML of *Stromatoneurospora phoenix* and other selected Xylariales based on ribosomal internal transcribed spacer (ITS) DNA sequence data. Support values of via MP, ML, and Bayesian (MB) analyses higher than 50% (MP, ML) and 0.95 (MB) and are given above (MP/ML) and below (MB) the respective branches. The black arrow indicates the sequences of *S. phoenix* (in orange font) and the clade comprising the sequences of the coprophilic and pyrophilic Xylariaceae is marked by a grey rectangle.

Aside from providing a full morphological description of the holomorph of this fungus, we also have generated DNA sequences of multiple loci for the first time, in addition to the analysis using ITS barcode. In MP analysis using only ITS rDNA, a CI of 0.376, RI of 0.461, and a HI of 0.624 and yielded five equally most parsimony trees with a length of 2097 changes. The phylogenetic relationships inferred from RAxML had a likelihood of −9550.538. The matrix had 485 distinct alignment patterns, with 28.28% undetermined characters or gaps. Estimated base frequencies were as follows: A = 0.239, C = 0.268, G = 0.252, T = 0.239; substitution rates AC = 1.764, AG = 3.446, AT = 2.371, CG = 1.583, CT = 4.927, GT = 1.000; gamma distribution shape parameter *α* 0.348. The likelihood of the Bayesian tree was −0.440. As shown in the ITS based phylogeny presented in Figure 3, the Thai strains of *S. phoenix* are not 100% identical with strain F-160,834 from Mexico (i.e., the only available sequence of this species in GenBank), differing at 16 base positions to each other. This may well be due to intragenomic polymorphisms of the ITS as recently found in several other species of Xylariales [39], or the Mexican fungus may actually constitute a different taxon. To verify that this is not due to intraspecies variation, which is common in fungi, a separate analysis using ITS, LSU, and other protein-coding genes (*RPB2*, *TUB2*) was conducted to see their phylogenetic affinities.

Aside from providing a full morphological description of the holomorph of this fungus, we also have generated DNA sequences of multiple loci for the first time. As shown in the ITS based phylogeny presented in Figure 3, the Thai strains of *S. phoenix* are not 100% identical with strain F-160,834 from Mexico (i.e., the only available sequence of this species in GenBank), differing at 16 base positions to each other. This may well be due to intragenomic polymorphisms of the ITS as recently found in several other species of Xylariales [39], or the Mexican fungus may actually constitute a different taxon. To verify that this is not due to intraspecies variation, which is common in fungi, a separate analysis using ITS, LSU, and other protein-coding genes (*RPB2*, *TUB2*) was conducted to assess their phylogenetic affinities.

**Figure 4:**
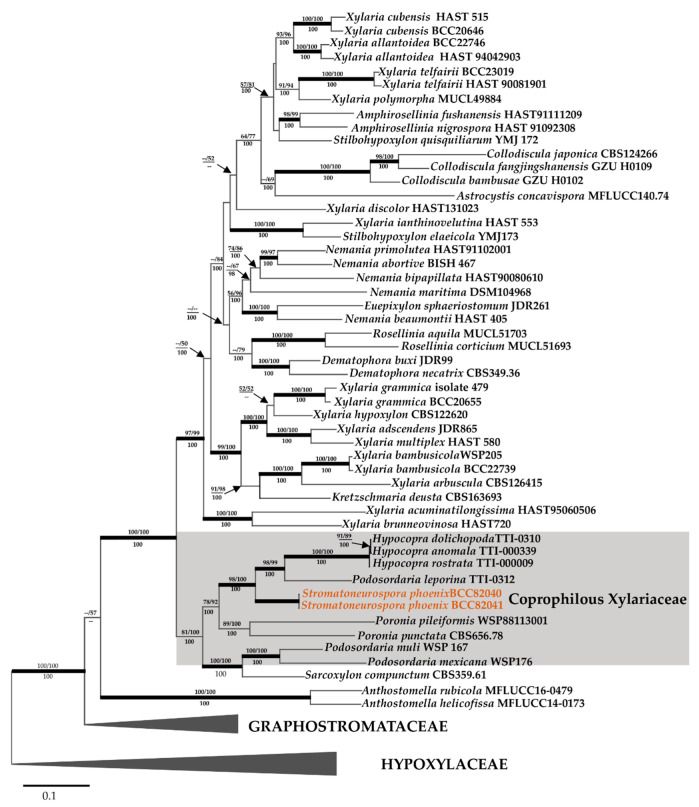
Phylogenetic relationships inferred from RAxML on multi-locus alignment of *Stromatoneurospora phoenix* and other selected Xylariales based on concatenated ribosomal (ITS and LSU) and proteinogenic (TUB2 and RPB2) DNA sequence data. Support values of via MP, ML and Bayesian (MB) analyses higher than 50% (MP, ML) and 0.95 (MB) and are given above (MP/ML) and below (MB) the respective branches. Branches of significant support (BS ≥ 95% and PP ≥ 0.95) are thickened. The black arrow indicates the sequences of *S. phoenix* (in orange font) and the clade comprising the sequences of the coprophilic and pyrophilic Xylariaceae is marked by a grey rectangle.

As shown in Figure 4, the 23 sequences that were newly generated from the combined ITS, LSU, *RPB2,* and *TUB2* data were compared with data from the public domain. This was done to clarify the phylogenetic relationships of newly collected Thai specimens of Xylariaceae and distinguish them from other species and genera in the stromatic Xylariales (PCR amplifications yielded approximately 500 bp, 1000 bp, 800 bp, and 1000 bp of ITS rDNA, LSU rDNA, *RPB2*, *TUB2* sequences, respectively). The phylogenetic relationships were estimated using the MP and ML analyses. The dataset of the multi-locus DNA sequences including 51 taxa in the Xylariaceae based on *Amphirosellinia* (2), *Anthostomella* (2), *Astrocystis* (1), *Collodiscula* (3), *Dematophora* (2), *Euepixylon* (1), *Hypocopra* (3), *Kretzschmaria* (1), *Nemania* (5), *Podosordaria* (3), *Poronia* (2), *Rosellinia* (2), *Sarcoxylon* (1), *Stilbohypoxylon* (2), *Stromatoneurospora* (2), and *Xylaria* (19). The combined dataset consisted of 4139 characters, of which 1882 were constant, 1668 parsimony informative, and 589 uninformative. In the MP analysis a CI of 0.305 a RI of 0.441 and a HI of 0.695 and yielded only one parsimony tree with a length of 14,069 changes. The phylogenetic relationships inferred from RAxML had a likelihood of −0.961. The matrix had 2453 distinct alignment patterns, with 31.97% undetermined characters or gaps. Estimated base frequencies were as follows: A = 0.238, C = 0.269, G = 0.252, T = 0.239; substitution rates AC = 1.388, AG = 4.244, AT = 1.383, CG = 1.153, CT = 5.674, GT = 1.000; gamma distribution shape parameter *α* 0.334. The likelihood of the Bayesian tree was −57,474.149.

As shown in Figure 4 in a grey rectangle, the sequences of the new Thai strains of *Stromatoneurospora phoenix* clustered with the Xylariaceae. As the topology of the phylogenetic tree is quasi identical to the one presented by Wendt et al. [4], from which the majority of DNA sequence data were derived using essentially the same methodology, we restrict our discussion on the phylogenetic position of the new data. The *Stromatoneurospora phoenix* sequences appeared distant from the various clades containing *Xylaria* and other xylariaceous genera as sister clade to the one comprising *Podosordaria leporina* and *Hypocopra*, and the other *Podosordaria* species studied along with *Sarcoxylon* appeared in another sister clade that was closely related to the one containing *Poronia*. Implications of these findings are discussed further below in the Discussion section.

### 3.3. Isolation and Structure Elucidation of Secondary Metabolites

In total, six compounds (**1**–**6**) were isolated from cultures of *Stromatoneurospora phoenix*, two of which constitute novel natural products (**1**–**2**) (Figure 5). All structures were elucidated by a combination of HR-ESI-MS as well as one- and two-dimensional NMR spectroscopy (see Figures S5–S18), assisted by UV/vis- and Electronic Circular Dichroism (ECD) Spectroscopy (Figures S3–S4) as well as Polarimetry.

**Figure 6:**
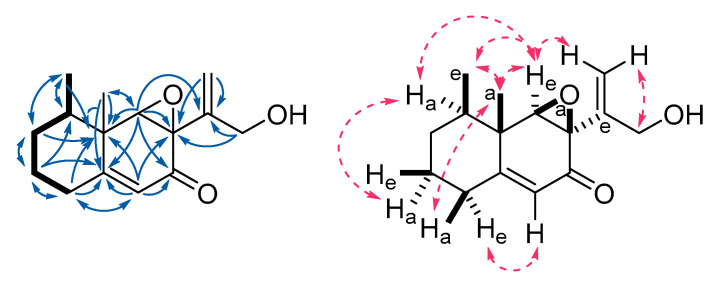
Key nuclear magnetic resonance (NMR) correlations of phoenixilane A (**1**). **Left**: structure with ^1^H/^1^H Correlation Spectroscopy-(COSY; bold bonds) and ^1^H/^13^C Heteronuclear Multiple Bond Correlation (HMBC) correlations (blue arrows). **Right**: relative conformation with Rotating Frame Nuclear Overhauser Effect Spectroscopy (ROESY) correlations (dashed, pink arrows); conformation of ring substituents: a: axial, e: equatorial.

Phoenixilane A (**1**) showed a molecular formula (MF) of C_15_H_2o_O_3_, as derived by High Resolution Electrospray Mass Spectrometry (HR-ESI-MS). Its ^1^H NMR and ^1^H/^13^C Heteronuclear Single Quantum Coherence Spectroscopy (^1^H/^13^C HSQC, Figure 6) spectra indicated presence of two methyls C-14 (doublet, d) and C-15 (singlet, s), one exomethylene C-13 (multiplet, m), one olefinic methine C-9 (s), three methylenes (C-1 to C-3, m), as well as two methines C-4 (m) and C-6 (s). One of the methylenes (C-12) and the methines (C-6) were strongly shifted downfield, respectively. The ^13^C NMR spectrum of **1** showed additional presence of one ketone, two sp^2^-, as well as four sp^3^-hybridized carbons carrying no protons. Analysis of the ^1^H/^1^H Correlation Spectroscopy (^1^H/^1^H COSY, Figure 6) spectra linked 1-H, 2-H_2_, 3-H_2_, 4-H_2_, and 14-H_3_. ^1^H/^13^C Heteronuclear Multiple-Bond Correlation Spectroscopy (^1^H/^13^C HMBC, Figure 6) correlations from 14-H_3_ to C-4, C-3, and C-5 showed that C-14 is bound to C-4. C-15, in turn, is linked to C-5, as proven by ^1^H/^13^C HMBC correlations of 15-H_3_ to C-4, C-5, and C-6. As 1-H_2_, 2-H_2_, and 15-H_3_ showed correlations to the sp^2^-hybridized C-10, a six-membered ring was indicated. ^1^H/^13^C HMBC correlations from the olefinic 9-H to C-10 (*δ*_C_ = 169.0 ppm) and C-8 (*δ*_C_ = 192.6 ppm) revealed the presence of an *α*,*β*-unsaturated ketone.

The chemical shifts of the methylene C-13 suggested an exomethylene functional group, with C-11 as the only remaining sp^2^-carbon to form the other part of the double bond. ^1^H/^13^C HMBC signals of 13-H_2_ to the hydroxy-carrying C-12 as well as C-7 fixed its position. As both C-6 and C-7 had chemical shifts indicating a link to an oxygen atom, but only one oxygen was left according to the MF of **1**, an epoxide ring was deduced. Presence of the epoxide was supported by the total number of six double bond equivalents (DBE) calculated for **1**, which can thus be assigned as follows: two six-membered rings (2 DBE), *α*,*β*-unsaturated ketone (2 DBE), exomethylene (1 DBE), epoxide (1 DBE).

The overall NMR analysis led to the identification of **1** as an unprecedented eremophilane sesquiterpenoid. The relative stereochemistry of **1** was assigned via Rotating Frame Nuclear Overhauser Effect Spectroscopy (ROESY) correlations. For the western cyclohexane ring, a chair conformation was derived by ROESY correlations between 2-H_axial_ and 4-H_a_ as well as 15-H_3a_ and 1-H_a_. For the eastern cyclohexanone moiety, a planar conformation was deduced. Occurrence of strong Rotating Frame Nuclear Overhauser Effect Spectroscopy (ROESY) correlations between the axial 15-H_3a_, as well as 14-H_3_ and 6-H indicated their vicinity and that both methyl groups 14-H_3 equatorial_ and 15-H_3a_ have a gauche-conformation. Furthermore, a correlation between 4-H_a_ and 6-H_e_ was observed, which too suggests a gauche-position. This allowed for the epoxide ring C-7/C-8 only to be attached axially and, in turn, rendering the prop-2-en-1-ol chain (C-11 to C-13) equatorially. These correlations are depicted as stereo and Newman projections in Figure S2. The absolute configuration of **1** was merely suggested to be 4*R*,5*S*,6*R*,7*R* by its specific optical rotation and electronic circular dichroism (ECD; Figure S3) spectrum in comparison with literature known compounds (Table S1), but needs further proof, e.g., by derivatization. 

Phoenixilane B (**2**) had a molecular formula of C_15_H_2o_O_4_, indicating a formal addition of one oxygen as compared to **1**. Most NMR signals and correlations of **1** were also found in the spectra of **2**, except for the lack of the exomethylene double bond C-11/C-13. Instead, C-11 was a shown to be a sp^3^-hybridized carbon linked to the methylene C-13, both of which form a second epoxide ring with the remaining oxygen atom. This was again supported by the number of six DBE present in **2**, i.e., the DBE that was assigned to the exomethylene in **1** was replaced by a DBE assigned to the second epoxide in **2**. The relative stereochemistry of C-4 to C-7 was deduced from ROESY data as done for **1.** Due to highly similar specific optical rotation values, ECD spectra (Figure S3), and biosynthetic origin, both are assumed to share the same backbone stereochemistry, i.e., absolute configuration, which is accordingly suggested to be 4*R*,5*R*,6*R*,7*S*. The stereochemistry of the second epoxide (C-11 and C-13) remains unclear.

In addition, the structures of four known metabolites were identified by comparison of the spectral data with references provided in the literature. These were (a) punctaporonin B (**3**), previously reported from *Poronia punctata* [40]; (b) 8,9-dehydroxylarone (**4**), previously isolated from a *Xylaria* sp. [41]; (c) (−)-(*R*)-6-hydroxy-3-methyl-4-dihydroisocoumarin-5-carboxylic acid (**5**), which was semi-synthetically prepared from 5-formylmellein [42] but never directly isolated and reported as a natural product before, and (d) 3-methoxycarbonyl indole (**6**), which has been reported from *X. cubensis* before [43]. The chemotaxonomic implications of these findings will be discussed further below.

### 3.4. Biological Activities

The antimicrobial effects of compounds **1**, **2**, and **4** against various bacterial and fungal test organisms were evaluated in a serial dilution assay. Only **2** showed inhibition of *Mucor hiemalis* at the highest concentration tested, i.e., 66.7 µg/mL (Table S2). No other antimicrobial activities were observed in the assay.

Furthermore, **2** exhibited weak cytotoxicity against the cell lines L929 (mouse fibroblasts) and KB 3.1 (human endocervical adenocarcinoma) at 31.1 and 68.2 µM, respectively (Table S3). No inhibition of viability was observed for compounds **1** and **4**. Due to the occurrence of cytotoxic activity in **2**, five additional human cell lines were examined**.** IC_50_ values for those ranged from 68.2 to 14.4 µM, with the lowest values measured against cell lines MCF-7 (14.4 µM) and A431 (17.4 µM).

## 4. Discussion

The phylogeny presented here is in accordance with previous hypotheses as inferred from morphological studies where the genera *Poronia*, *Podosordaria*, *Sarcoxylon*, and *Stromatoneurospora* had been believed to have affinities to *Xylaria* because of the “centrum structure” of their ascomata. They differ from typical *Xylaria* by lacking the strong melanization of their stromata, and *Poronia* and *Podosordaria* have different anamorph types [3]. The fact that a lindquistia-like conidial state was observed in *S. phoenix* in the current study is actually in accordance with the phylogenetic data. The genus *Lindquistia* was once erected to accommodate the anamorph of *Podosordaria leporina* [44], and Rogers already had pointed out in 1985 that the lindquistia-like conidiophores are commonly encountered in both *Podosordaria* and *Poronia* [45].

While the ecology of the genus *Sarcoxylon*, whose stromata have only been collected from wood, still needs further study, *Poronia* and *Podosordaria* were traditionally separated from *Xylaria* also because of their coprophilic lifestyle. On the other hand, *Stromatoneurospora* is regarded as a pyrophilic genus as it has almost exclusively been collected from burnt Poaceae. It is believed that fire heat-activates ascospores in the grass rhizosphere. Alternatively, the fungus may be an endophyte that forms stromata associated with sexual reproduction when the host is damaged, increasing the chances of infecting new host plants via ascospores. There are several examples of stromatic Xylariales where endophytism has been proven, such as *Daldinia vernicosa* and other species of that genus, which are definitely endophytes and form their stromata when the host plant is burnt [28]. However, we did not find any similar sequences in GenBank when using the ITS data of *S. phoenix* in a BLAST search among the environmental sequence data. The closest match was the ITS sequence of *Areolospora bosensis* resulting from the study by Vu et al. [33] with less than 95% similarity. The potential relationships between non systemic fungal endophytes and coprophilic fungi have been discussed by Marquez et al. [46], who did, however, not specifically refer to the Xylariaceae. On the other hand, in the Sordariomycetes, and in particular the Sordariales there are several other examples of genera and families containing both, coprophilic and pyrophilic species [47]. By now, it is well-known that the Xylariaceae and many other families of Sordariomycetes are very rich in endophytes [48] but also contain some coprophilic lineages. During early investigations of endophytes in the 20^th^ century, when molecular phylogenetic methods were not yet available and mycologists needed to rely on culturing techniques and careful morphological studies, it was suggested that the coprophilic habit may be rather transitional stage in the life cycle of the coprophilic fungi [49]. They must be able to survive in the soil or on plant surfaces after the nutrient-rich substrata have been exhausted, and they are in strong competition with other organisms that co-exist in the coprophilic habitat. In comparison to saprotrophs that colonize dead wood, they must reproduce relatively fast and quickly exploit a more limited carbon base. The same is true for the pyrophilic fungi, which also have to colonize the burnt substrata quickly [50]. We thus speculate that having a diminutive stroma (as in *Podosordaria*), or a stroma essentially reduced to a subiculum (as in *Poronia* and *Hypocopra*) is a morphological/reproductive adaptation. On the other hand, the horizontally transmitted endophytes, to which most of the genera of Xylariaceae obviously belong, are “inducible mutalists” [51]. They can also persist outside the host plant and grow rapidly under favorable conditions, but may remain dormant and metabolically inactive for a long time if they are hidden in a healthy host plant. If the host is totally incinerated, they will hardly have any chance to escape, but many species (including several *Daldinia* spp. [28]) may occur on semi-burnt wood or on trees that were hit by lightning, and form their stromata shortly after the damage to the host has occurred. Nevertheless, their life strategy is much different from that or a coprophilic or pyrophilic fungus.

On the other hand, several kinds of coprophilic fungi, e.g., *Preussia* (=*Sporormiella*), and *Delitschia* species, have been reported as endophytes from surface-disinfected (often incorrectly referred to in the literature as “surface-sterilized” plant tissues). There are occasional reports in the literature, suggesting that the coprophilic fungi can persist in plant material, even including traditional herbal drugs [52]. There are also some early systematic studies that show that “typical” coprophiles like *Sordaria fimicola* and *Sporormiella* spp. do not occur in the xylem of the studied host plants but are restricted to the outer bark [53], whereas Xylariales and other Sordariomycetes that inhabit the same plant occur preferentially in the xylem or may be either endophytic or epiphytic. Even though these studies do not concern Xylariales, they might explain why we were unable to detect an ITS sequence similar to *Stromatoneurospora* and the coprophilic Xylariaceae genera among the sequences derived from endophytes and environmental plant material in GenBank.

In some cases, in the Xylariaceae itself, evidence from molecular phylogenies is now increasing that the coprophilic genera have developed as an independent evolutionary lineage from their wood-inhabiting, endophytic relatives. In the phylogenetic tree depicted in Figure 4, the genera *Hypocopra*, *Poronia* and *Podosordaria* cluster together with *Sarcoxylon* as the only “non-copro-/pyrophilic” genus that is thus far only known to inhabit wood. The phylogenetic affinities of the latter genus can be explained by morphological characters, but little is known about the ecology of the rarely collected species of *Sarcoxylon*, which are only known from very few specimens that were scattered around the tropics [54]. The only coprophilic xylariaceous genus of which no DNA sequence data are available as now is actually *Wawelia*, which deviates from the other genera by having a characteristic geniculosporium-like anamorph but its stromata are also lacking the typical melanization that is typical of *Xylaria* and its immediate allies [4].

To our knowledge, this is the first confirmed record of *S. phoenix* from Thailand and Asia, even though it seems to have a circumtropical distribution. It has been recorded before from Vietnam (specimen housed in the natural history museum, Stockholm, with acc. No F145680, without any details on collector and mode of identification) and Australia (Queensland, Cape York Peninsula, near Heathlands Ranger Station, at ground level on remnants of dead monocotyledon leaf and stem in sandy soil. 23 March 1992; leg, Cribb, A.B. and J.W. 20686, deposited as BRIP 20686) according to the GBIF database [55]. During their type studies on *Stromatoneurospora phoenix* and other xylarialean fungi with aberrant ascospore morphology, Rogers et al. [56] have also tentatively referred to specimens from Africa (Uganda) and India that were previously treated as *Xylaria kurziana* and from New Caledonia, previously treated as *Xylaria ustorum*, as well as on a specimen from Hawaii. All other records of this species are from the neotropics or the southern USA. As the old herbarium specimens were not in good conditions, further fieldwork in the tropics should be carried out in these geographic areas to obtain and culture fresh material of these fungi in order to clarify whether they belong to *Stromatoneurospora phoenix* or constitute additional members of this genus.

The isolated eremophilane sesquiterpenoids phoenixlanes A–B (**1**–**2**) constitute unprecedented structures. While compound **1** did not show antimicrobial or cytotoxic activities, compound **2** exhibited weak cytotoxic effects against mammalian cells lines with the highest activity against MCF-7 cells (IC_50_ of 14.4 µM). Several eremophilanes have been described already from related fungi like *Xylaria* spp. [57,58,59] or *Podosordaria tulasnei* [60], but further studies of eremophilanes from relatives of *S. phoenix* need to be conducted to verify their chemotaxonomic potential.

Of the secondary metabolites isolated, compounds **3**–**4** and **6** have some chemotaxonomic significance because they were previously known from the stromatic Xylariales. While compound **4** and related pyrones are known from a wide range of xylarialean fungi and can even be found in various other ascomycetes, compound **6** has been reported from canola roots [61], but also from the related *Xylaria cubensis* [43]. The punctaporonins, such as compound **3**, however, were so far only found in the genus *Poronia*, which appeared in our phylogeny as closest relative to *Stromatoneurospora*. These caryophyllene sesquiterpenoids may turn out to be valid chemotaxonomic markers once more strains and species of *Stromatoneurospora,* and allied genera, have been cultured and examined for their presence.

## 5. Conclusions

The current study has shed light on the affinities of an interesting pyrophilic xylarialean fungus and a combination of morphological, chemotaxonomic, and molecular data has clearly revealed its closest relatives. The suspicions by Rogers [62], who based his concept on morpho-anatomical studies of the stromata and ascospores that *Stromatoneurospora* is a relative of the coprophilous Xylariaceae were thereby confirmed after 40 years. To strengthen the taxonomic position of *S. phoenix* and its close relatives, material from South America, (i.e., the geographic area where *Stromatoneurospora* has been first reported) should be collected, cultured, and used for epitypification.

## Figures and Tables

**Figure 5:**
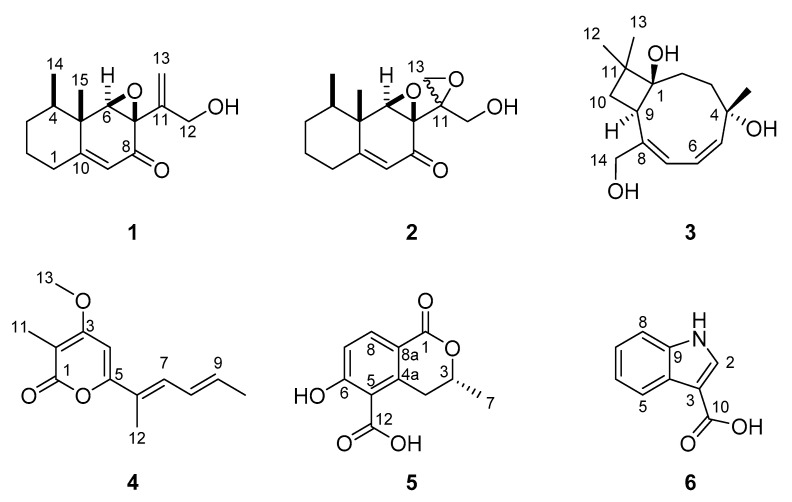
Chemical structures of secondary metabolites isolated from cultures of *Stromatoneurospora phoenix*. Phoenixilanes A–B (**1**,**2**) punctaporonin B (**3**), 8,9-dehydroxylarone (**4**), (−)-(*R*)-6 hydroxy-3-methyl-4-dihydroisocoumarin-5-carboxylic acid (**5**), and 3-methoxycarbonyl indole (**6**).

**Table 1:** List of all taxa used in the current study. **ET (boldface)**: epitype strain, **HT (boldface)**: holotype strain, **PT (boldface)**: paratype strain.

Taxon	Strain/Status	Country	GenBank Accession Numbers				Reference
			ITS	LSU	*RPB2*	*TUB2*	
*Amphirosellinia fushanensis*	HAST91111209/**HT**	Taiwan	GU339496	N/A	GQ848339	GQ495950	[23]
*Amph. nigrospora*	HAST 91092308/**HT**	Taiwan	GU322457	N/A	GQ848340	GQ495951	[23]
*Anthostomella helicofissa*	MFLUCC14-0173	Italy	KP297406	N/A	KP340534	KP406617	[3]
*Anth. rubicola*	MFLUCC16-0479	Italy	KX533455	KX533456	N/A	N/A	[3]
*Astrocystis concavispora*	MFLUCC140.74	Italy	KP297404	KP340545	KP340532	KP406615	[3]
*Biscogniauxia nummularia*	MUCL 51395/**ET**	France	KY610382	KY610427	KY624236	KX271241	[4]
*Collodiscula bambusae*	GZU H0102	China	KP054279	KP054280	KP276675	KP276674	[24]
*C. fangjingshanensis*	GZU H0109/**ET**	China	KR002590	KR002591	KR002592	KR002589	[24]
*C. japonica*	CBS124266	China	JF440974	JF440974	KY624273	KY624316	ITS: [25]; LSU: [4]
*Daldinia concentrica*	CBS 113277	Germany	AY616683	KY610434	KY624243	KC977274	ITS: [26]; *TUB2*: [27]; [4]
*Dematophora necatrix*	CBS349.36	Argentina	AY909001	KF719204	KY624275	KY624310	ITS: [5]; LSU: [4]
*De. buxi*	JDR99	France	GU300070	N/A	GQ844780	GQ470228	[23]
*Euepixylon sphaeriostomum*	JDR261	USA	GU292821	N/A	GQ844774	GQ470224	[23]
*Graphostroma platystomum*	CBS 270.87	France	JX658535	DQ836906	KY624296	HG934108	ITS: [28]; LSU: [29]; *RPB2*: [30]; *TUB2*: [4]
*Hypocopra anomala*	TTI-000339	in press	N/A	MT903245	MT901033	MT901030	this study
*Hypoc. dolichopoda*	TTI-0310	USA	N/A	MT903247	MT901035	N/A	this study
*Hypoc. rostrata*	TTI-000009	USA	MT896134	MT903246	MT901034	MT901031	this study
*Hypoxylon fragiforme*	MUCL 51264/**ET**	Germany	KC477229	KM186295	KM186296	KX271282	ITS: [31]; *RPB2*: [3,4]
*Kretzschmaria deusta*	CBS163693	Germany	KC477237	KY610458	KY624227	KX271251	[4]
*Nemania abortiva*	BISH 467/**HT**	USA	GU292816	N/A	GQ844768	GQ470219	[23]
*Nem. beaumontii*	HAST405	Martinique	GU292819	N/A	GQ844772	GQ470222	[23]
*Nem. bipapillata*	HAST90080610	Taiwan	GU292818	N/A	GQ844771	GQ470221	[23]
*Nem. maritima*	DSM104968	France	KY610414	KY610414	N/A	N/A	[4]
*Nem. primolutea*	HAST91102001/**HT**	Taiwan	EF026121	N/A	GQ844767	EF025607	[23]
*Podosordaria leporina*	TTI-0312	USA	N/A	MT903244	MT901032	MT901029	this study
*Podos. mexicana*	WSP176	Mexico	GU324762	N/A	GQ853039	GQ844840	[23]
*Podos. muli*	WSP 167/**HT**	Mexico	GU324761	N/A	GQ853038	GQ844839	[23]
*Poronia pileiformis*	WSP88113001/**ET**	Taiwan	GU324760	N/A	GQ853037	GQ502720	[23]
*Poronia punctata*	CBS656.78	Australia	KT281904	KY610496	KY624278	KX271281	ITS: [32]; [4]
*Rosellinia aquila*	MUCL51703	France	KY610392	KY610460	KY624285	KX271253	[4]
*Ros. corticium*	MUCL51693	France	KY610393	KY610461	KY624229	KX271254	[4]
*Sarcoxylon compunctum*	CBS359.61	South Africa	KT281903	KY610462	KY624230	KX271255	ITS: [32]; [4]
*Stilbohypoxylon elaeidicola*	YMJ173	French Guiana	EF026148	N/A	GQ844826	EF025616	[23]
*Stilboh. quisquiliarum*	YMJ 172	French Guiana	EF026119	N/A	GQ853020	EF025605	[23]
*Stromatoneurospora phoenix*	BCC82040	Thailand	MT703666	MT735133	MT742605	MT700438	this study
*Stromatoneurospora phoenix*	BCC82041	Thailand	MT703667	MT735134	MT742606	MT700439	this study
*Stromaton. phoenix*	F-160, 834	Mexico	AY909004	N/A	N/A	N/A	[5]
*Stromaton*. *phoenix*	OTU_33	China	MH430290	N/A	N/A	N/A	[6]
*Xylaria acuminatilongissima*	HAST95060506/**HT**	Taiwan	EU178738	EU178738	EU178738	EU178738	[23]
*Xyl. adscendens*	JDR 865	Thailand	GU322432	N/A	GQ844818	GQ487709	[23]
*Xyl. allantoidea*	HAST 94042903	Taiwan	GU324743	N/A	GQ848356	GQ502692	[23]
*Xyl. allantoidea*	BCC22746	Thailand	MT703671	MT735141	MT742610	N/A	this study
*Xyl. arbuscula*	CBS126415	Thailand	MH864101	KY610463	KY624287	KX271257	ITS: [33]; LSU, *RPB2*, *TUB2*: [4]
*Xyl. bambusicola*	BCC22739	Thailand	MT710944	MT735135	MT742614	N/A	this study
*Xyl. bambusicola*	WSP205/**HT**	Thailand	EF026123	N/A	GQ844802	AY951762	[23]
*Xyl. brunneovinosa*	HAST720/**HT**	Taiwan	EU179862	N/A	GQ853023	GQ502706	[23]
*Xyl. cubensis*	HAST 515	Martinique	GU373810	N/A	GQ848366	GQ502701	[23]
*Xyl. cubensis*	BCC20646	Thailand	MT703672	MT735142	MT742611	N/A	this study
*Xyl discolor*	HAST131023	USA	JQ087405	N/A	JQ087411	JQ087414	[34]
*Xyl. grammica*	BCC20655	Thailand	MT703670	MT735138	MT742609	N/A	this study
*Xyl. grammica*	isolate 479	Taiwan	GU300097	N/A	GQ844813	GQ487704	[23]
*Xyl. hypoxylon*	CBS122620/**ET**	Sweden	KY610407	KY610495	KY624231	KX271279	[23]
*Xyl. ianthinovelutina*	HAST 553	Martinique	GU322441	N/A	GQ844828	GQ495934	[23]
*Xyl. multiplex*	HAST 580	Martinique	GU300098	N/A	GQ844814	GQ487705	[23]
*Xyl. polymorpha*	MUCL49884	France	KY610408	KY610464	KY624288	KX271280	[4]
*Xyl. telfairii*	BCC23019	Thailand	MT703674	MT735139	MT742613	N/A	this study
*Xyl. telfairii*	HAST 90081901	Thailand	GU324738	N/A	GQ848351	GQ502687	[23]

**Table 2:** One-dimensional (1D) NMR data of phoenixilanes A–B (**1**–**2**) (**1**: acetone-*d_6_*, **2**: DMSO-*d*_6_; ^1^H NMR: 500 MHz, ^13^C NMR: 125 MHz).

Pos ^1^	1		2	
	*δ*_C_, Mult ^2^	*δ*_H_, Mult ^2^	*δ*_C_, Mult ^2^	*δ*_H_, Mult ^2^
1	33.5, CH_2_	2.37, m 2.19, m	32.1, CH_2_	2.28, m 2.16, m
2	29.7, CH_2_	1.99, m 1.41, m	28.6, CH_2_	1.92, m 1.31, m
3	31.1, CH_2_	1.62, m	29.7, CH_2_	1.53, m
4	41.1, CH	1.86, m	39.8, CH	1.69, m
5	42.4, C		40.9, C	
6	70.3, CH	3.43, s	65.2, CH	3.58, s
7	62.8, C	-	59.9, C	-
8	192.6, C	-	191.4, C	-
9	120.6, CH	5.69, s	119.3, CH	5.72, s
10	169.0, C	-	169.1, C	-
11	146.7, C	-	58.3, C	-
12	63.7, CH_2_	4.26, d 4.17, d	60.4, CH_2_	3.42, ddd (11.90, 4.88, 0.70)
13	112.2, CH_2_	5.22, q (1.37) 5.13, dt (1.91, 1.03)	46.4, CH_2_	2.65, dd (11.98, 4.81) 2.55, dd (5.49, 0.61)
14	15.6, CH_3_	1.05, d (6.71)	15.0, CH_3_	0.98, d (6.71)
15	15.5, CH_3_	1.28, s	14.9, CH_3_	1.18, s
12-OH	-	-	-	4.88, dd (7.32, 4.88)

^1^ pos: atom position (see Figure 5); ^2^ *δ*_C_/*δ*_H_: chemical shift [ppm]; mult: multiplicity; s: singlet, d: doublet, t: triplet, m: multiplet.

## Supplementary Materials

The following are available online at https://www.mdpi.com/2309-608X/6/3/144/s1, Figure S1: HPLC-UV/vis chromatograms at 210 nm, DAD and HR-ESI-MS(+) traces of phoenixilanes A–B; Figure S2: Stereo-(top) and Newman projections (bottom) of phoenixlane A (**1**) with observed ROESY correlations for two possible relative conformations. Figure S3: UV/vis spectra of phoenixilanes A–B (**1**–**2**) from 200–500 nm. Figure S4: ECD spectra of phoenixilanes A–B (**1**–**2**) from 190–500 nm. Figure S1: ^1^H NMR spectrum (500 MHz, acetone-*d_6_*) of phoenixilane A (**1**). Figure S2: ^13^C NMR spectrum (125 MHz, acetone-*d_6_*) of phoenixilane A (**1**). Figure S3: ^1^H/^1^H COSY spectrum (500 MHz, acetone-*d_6_*) of phoenixilane A (**1**). Figure S4: ^1^H/^13^C HSQC spectrum (500 MHz, acetone-*d_6_*) of phoenixilane A (**1**). Figure S9: 1H/13C HMBC spectrum (500 MHz, acetone-d6) of phoenixilane A (**1**). Figure S5: ^1^H/^1^H ROESY spectrum (500 MHz, acetone-*d_6_*) of phoenixilane A (**1**). Figure S6: Zoomed ^1^H/^1^H ROESY spectrum (500 MHz, acetone-*d_6_*) of phoenixilane A (**1**). Figure S7: 1H NMR spectrum (500 MHz, DMSO-*d_6_*) of phoenixilane B (**2**). Figure S8: ^13^C NMR spectrum (125 MHz, DMSO-*d_6_*) of phoenixilane B (**2**). Figure S9: ^1^H/^1^H COSY spectrum (500 MHz, DMSO-*d_6_*) of phoenixilane B (**2**). Figure S10: ^1^H/^13^C HSQC spectrum (500 MHz, DMSO-*d_6_*) of phoenixilane B (**2**). Figure S11: ^1^H/^13^C HMBC spectrum (500 MHz, DMSO-*d_6_*) of phoenixilane B (**2**). Figure S12: ^1^H/^1^H ROESY spectrum (500 MHz, DMSO-*d_6_*) of phoenixilane B (**2**). Figure S13: Zoomed ^1^H/^1^H ROESY spectrum (500 MHz, DMSO-*d_6_*) of phoenixilane B (**2**). Table S1**:** Comparison of Specific Optical Rotations [α]D and Electronic Circular Dichroism (ECD) maxima/minima of phoenixilanes A–B (1–2) with literature-known structures**.** Table S2. Antimicrobial activities of phoenixilanes A–B (**1**–**2**) and 8,9-dehydroxylarone (**4**) as minimum inhibitory concentrations (MIC). Table S3. Cytotoxicities of phoenixilanes A–B (**1**–**2**) and 8,9-dehydroxylarone (**4**) as half maximal inhibitory concentrations (IC_50_).
