# Supplementary material for: Phylogenetic and Chemotaxonomic Studies Confirm the Affinities of Stromatoneurospora phoenix to the Coprophilous Xylariaceae
Source: J Fungi (Basel). 2020 Aug 23;6(3):144. doi: 10.3390/jof6030144 (PMC7558325; doi:10.3390/jof6030144)
Supplement: Supplementary file 1 [file jof-06-00144-s001.pdf]

Supporting Information

Phylogenetic and Chemotaxonomic Studies Confirm the Affinities of *Stromatoneurospora phoenix* to the Coprophilous Xylariaceae

Kevin Becker [1,2], Sarunyou Wongkanoun [3,4], Anna-Charleen Wessel [1], Gerald F. Bills [5], Marc Stadler [1,2,*], J. Jennifer Luangsa–ard [6,*]

1 Department of Microbial Drugs, Helmholtz Centre for Infection Research GmbH (HZI), Inhoffenstraße 7, 38124 Braunschweig, Germany; kevin.becker@helmholtz-hzi.de (K.B.), a.wessel@tu-bs.de (A.-C.W.)

2 German Centre for Infection Research Association (DZIF), partner site Hannover-Braunschweig, Inhoffenstraße 7, 38124 Braunschweig, Germany

3 Faculty of Biotechnology, College of Agricultural Innovation, Biotechnology and Food, Rangsit University, Phahonyothin Road, Rak–hok, Pathum Thani 12000, Thailand ; sarunyou.wong@gmail.com (S.W.)

4 National Biobank of Thailand (NBT), National Science and Technology Development Agency (NSTDA), 111 Thailand Science Park, Phahonyothin Road, Khlong Nueng, Khlong Luang, Pathum Thani 12120, Thailand ;

5 Texas Therapeutics Institute, The Brown Foundation Institute of Molecular Medicine, The University of Texas Health Science Center at Houston, 1881 East Road, Houston, TX 77054, USA ; billsge@vt.edu (G.B.)

6 Plant Microbe Interaction Research Team (APMT), Integrative Crop Biotechnology and Management Research Group, National Center for Genetic Engineering and Biotechnology (BIOTEC), 113 Thailand Science Park, Phahonyothin Road, Khlong Nueng, Khlong Luang, Pathum Thani 12120, Thailand; jajen@biotec.or.th (J.J.L.)

* Correspondence: marc.stadler@helmholtz-hzi.de (M.S.); Tel.: +49-531-6181-4240; Fax: +49-531-6181-9499; jajen@biotec.or.th (J.J.L.); Tel.: +66 2 56467003349, Fax:+66 2 5646707

Contents

Isolation Procedures of known Compounds **3**–**6** 2

HPLC-DAD-MS Chromatograms of Phoenixilanes A–B (**1**–**2**) 3

Stereo- and Newman Projections of Phoenixilane A (**1**) 4

ECD and UV/Vis Spectra of Phoenixilanes A–B (**1**–**2**)........ 5

1D and 2D NMR Spectra of Phoenixilane A (**1**) 7

1D and 2D NMR Spectra of Phoenixilane B (**2**)........ 19

Antimicrobial and Cytotoxic Activities of Phoenixlanes A–B (**1**–**2**) and 8,9-Dehydroxylarone (**4**) 28

MUSCLE Alignments of ITS, LSU, RPB2, TUB2 gene regions from *Stromatoneurospora phoenix* 28

Isolation Procedures of known Compounds 3–6

*Isolation of **3** and **5**–**6** from Submerged YM 6.3 Medium*

Separation of the YM 6.3 crude extract (2×380 mg, 5 mL of acetone per injection) was achieved using the Gilson PLC 2250 device and conditions as for the ZM ½ crude extract (solvent A: H_2O+0.1% formic acid, solvent B: ACN+0.1% formic acid; flow: 50 mL/min, *cf.* manuscript). This yielded the following, yet impure, samples: fraction I (43 mg, t_R = 32–33.5 min), II (34.6 mg, t_R = 35.5–38 min], and III (11.3 mg, t_R = 38.5–41.5 min).

Fraction I (43 mg) was further purified on the Gilson PLC 2250 system using the same eluents as before and a Nucleodur C18ec column (250×21 mm, 5 µm; SN 762022.210, Macherey-Nagel, Düren, Germany). The gradient conditions were as follows: flow rate 20 mL/min; 20% B for 5 min, increase to 50% B in 30 min, further increase to 100% B in 5 min, followed by isocratic conditions at 100% B for 10 min. This yielded 10.0 mg of punctaporonin B (**3**; t_R = 12–23 min)

Fraction II (34.6 mg) was further separated on the Gilson PLC 2250 system using the same eluents as before and a X-Bridge C18 column (250×19 mm, 5 µm; SN 186004021, Waters Corp., Milford, MA, USA). The gradient conditions were as follows: flow rate 20 mL/min; 10% B for 10 min, increase to 40% B in 35 min, further increase to 100% B in 5 min, followed by isocratic conditions at 100% B for 10 min. This yielded 7.4 mg of (−)-(*R*)-6-hydroxy-3-methyl-4-dihydroisocoumarin-5-carboxylic acid (**5**; t_R = 30–38.5 min)

Fraction III (11.3 mg) was further purified *via* preparative thin layer chromatography (TLC) using SILGUR UV254 glass plates (200×200 mm, 0.25 mm silica layer thickness; SN 810023, Macherey-Nagel). As eluent, 150 mL of dichloromethane DCM:acetone 9:1 were used. The sample (11.3 mg) was dissolved in 400 µL acetone:MeOH 3+1. This yielded 0.6 mg of 3-methoxycarbonylindole (**6**; R_f = 0.48–0.56)

*Isolation of **4** from Solid BRFT Medium*

The crude extract of the solid BRFT medium was dispersed in *ca*. 5 mL of H_2O and loaded onto an open RP solid phase cartridge (Strata® X 33 µm Polymeric Reversed Phase Tube, 1 g/12 mL; SN 8B-S100-JDG, Phenomenex, Aschaffenburg, Germany). Elution was achieved by using low vacuum and a step-gradient of H_2O:ACN:MeOH (each with 0.1% of formic acid) with the following steps of 40 mL: 100:0:0, 90:10:0, 60:40:0, 30:70:0, 0:100:0, 0:0:100. The effluents of all steps were dryed *in vacuo* at 40 °C and analysed by ESI-MS. Accordingly, the gradient step 30:70:0 (fraction IV, ca. 200 mg) was further processed.

The fraction IV was then subjected to the Gilson PLC 2250 system and Nucleodur C18ec column (125×40, 7 µm; SN 762042.400, Macherey-Nagel) with eluents and flow rate as mentioned before, but using a different gradient: isocratic conditions at 15% B for 3 min, followed by an increase to 65% B in 50 min, then increase to 100% B in 10 min, followed by isocratic conditions of 100% B for 10 min. This yielded the following, yet impure, fraction V (35.8 mg, t_R = 39.5–43 min).

Fraction V was then purified using the PLC 2250 system equipped with a Luna® C18 column (250×21 mm, 5 µm; SN 00D-4252-P0-AX, Phenomenex). Fractionation was set to 10 mL per fraction. The following gradient was applied: isocratic conditions at 35% B for 5 min, followed by an increase to 65% B in 30 min, then increase to 100% B in 5 min, followed by isocratic conditions at 100% B for 10 min. This yielded 12.6 mg of 8,9-dehydroxylarone (**4**; t_R = 23.5–25.5 min).

HPLC-DAD-MS Chromatograms of Phoenixilanes A−B (1−2)

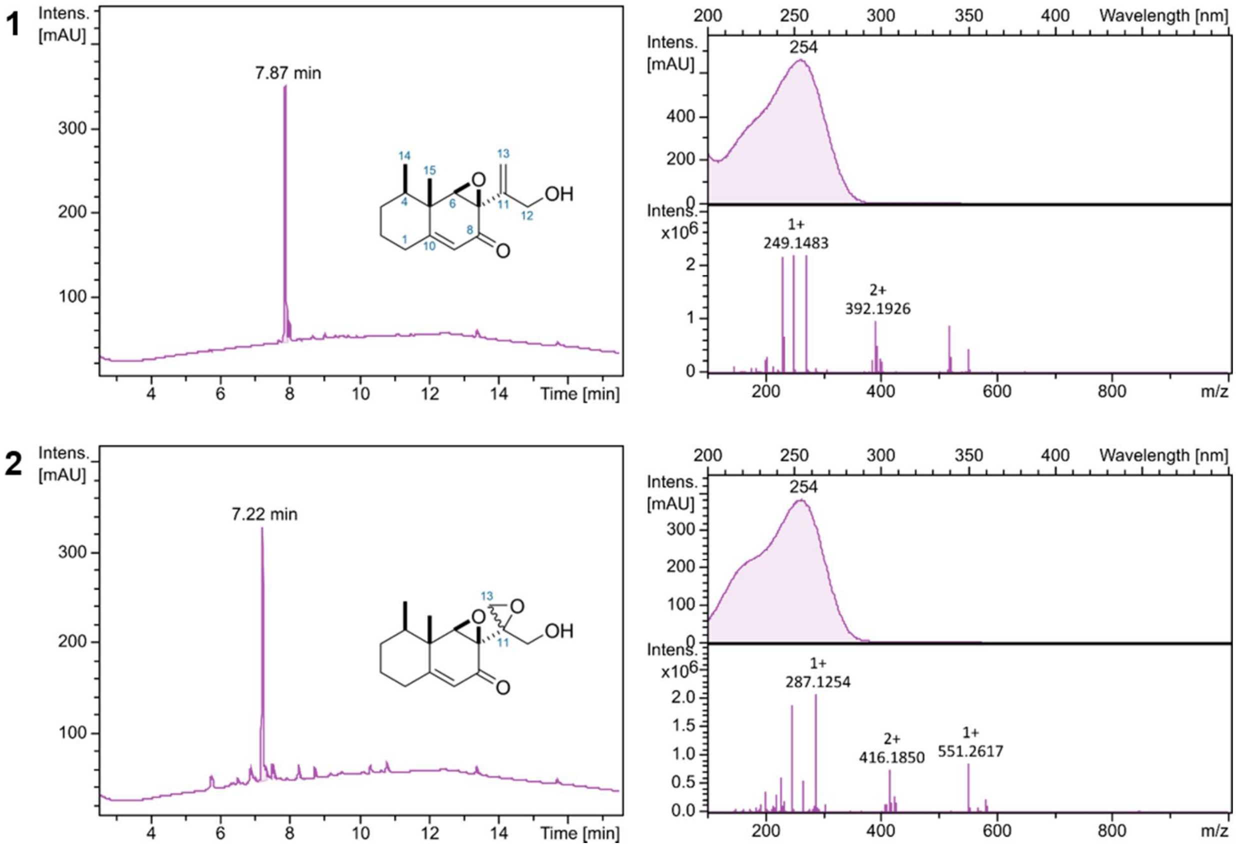

Figure S1: HPLC-UV/vis chromatograms at 210 nm, DAD and HR-ESI-MS(+) traces of phoenixilanes A−B (**1**−**2**).

Stereo- and Newman Projections of Phoenixilane A (1)

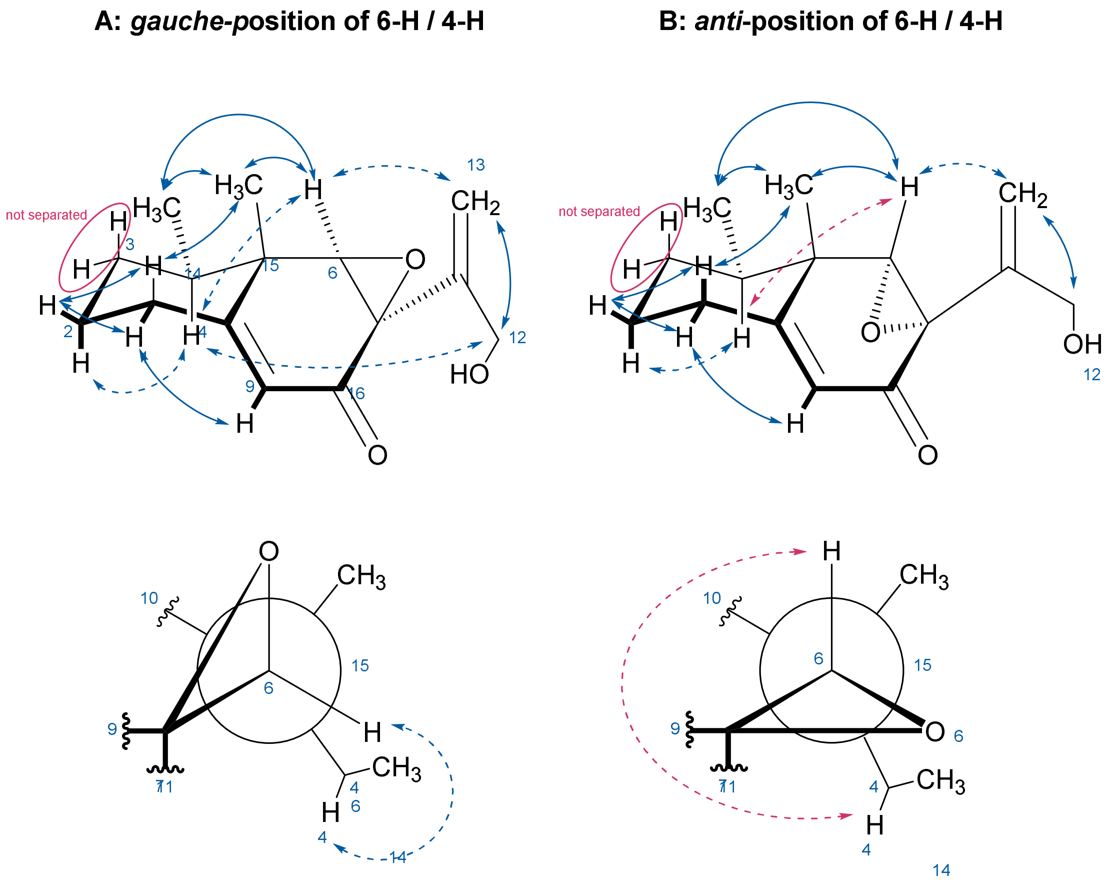

Figure S2: Stereo- (top) and Newman projections (bottom) of phoenixlane A (**1**) with observed ROESY correlations for two possible relative conformations (see Figures S10–S11). **A**: *gauche*-position of 6-H and 4-H , **B**: with an *anti*-position. Arrows indicate observed ROESY signals. Arrow type: solid: strong signals; dashed: weak signals. Arrow colours: blue: possible ROESY correlations in the respective conformation; pink: impossible ROESY signals (*e.g.* due to an *anti*-position of both protons). Occurrence of the ROESY correlation between 6-H and 4-H resulted in conformation **B** to be rejected.

ECD and UV/Vis Spectra of Phoenixilanes A−B (1−2)

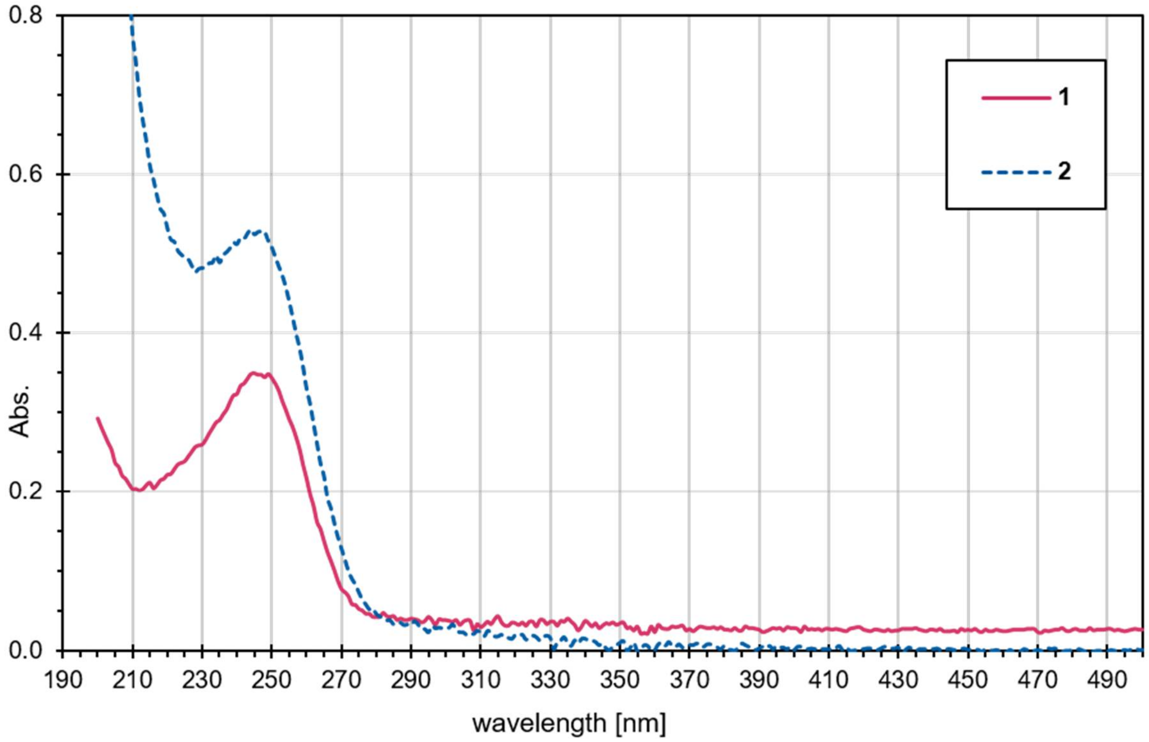

Figure S3: UV/vis spectra of phoenixilanes A−B (**1**−**2**) from 200−500 nm.

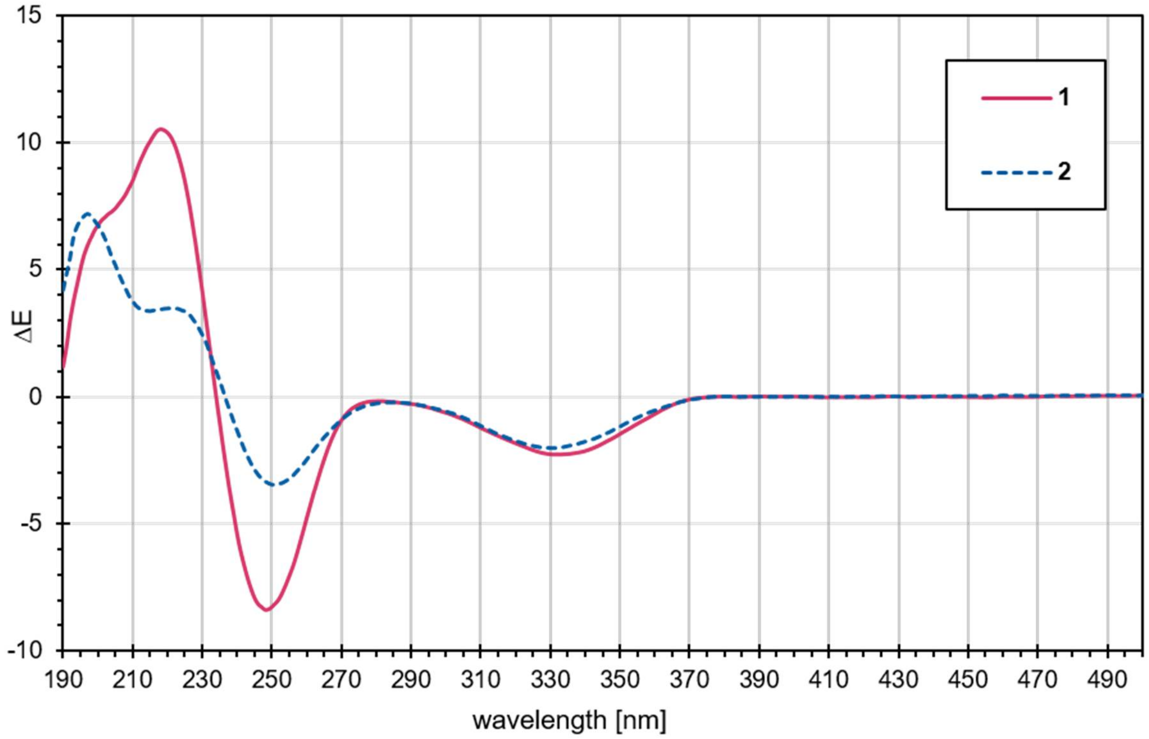

Figure S4: ECD spectra of phoenixilanes A−B (**1**−**2**) from 190−500 nm.

Table S1: Comparison of Specific Optical Rotations [*α*]D and Electronic Circular Dichroism (ECD) Cotton Effects (CE) of phoenixilanes A–B (**1**–**2**) with literature-known structures. For **1**–**2**, the suggested absolute configuration is depicted, while for the other compounds, the absolute stereochemistry as reported in the respective reference is shown.

	**Phoenixilanes**		**Peribysins**				**Miscellaneous**	
	**A**	**B**	**A**	**Q**	***ent*-Q**	**Intermediate product** [2]	**Phomadecalin A**	**Phomenol**
	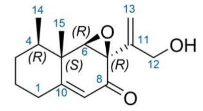	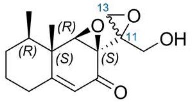	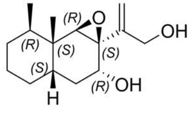	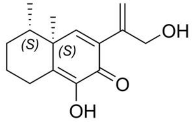	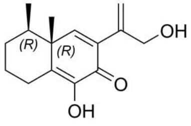	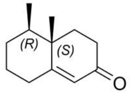	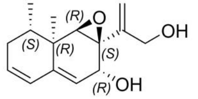	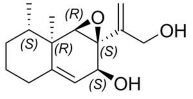
reference	this work	this work	[1]	[2]	[2]	[3]	[4]	[5]
origin	*S. phoenix*	*S. phoenix*	*Periconia byssoides*	*P. macrospinosa*	semisynthetic	semisynthetic	*Phoma* sp.	*Chrysoporthe* sp.
[*α*]D	−77	−54	−63.7	+18	−13.2	−163.2	+58	+23.3
ECD pos. CE [1] [nm]	218, 281	197, 221, 285	n/a	238, 332	300	n/a	n/a	n/a
ECD neg. CE [1] [nm]	248, 331	215, 251, 330	n/a	300	237, 333	n/a	n/a	n/a

[1] CE: cotton effect; [2] Intermediate product IUPAC name: (4a*S*,5*R*)-4a,5-dimethyl-4,4a,5,6,7,8-hexahydronaphthalen-2(3*H*)-one

1. Yamada, T.; Iritani, M.; Minoura, K.; Kawai, K.; Numata, A. Peribysins A–D, potent cell-adhesion inhibitors from a sea hare-derived culture of Periconia species. *Org. Biomol. Chem.* **2004**, *2*, 2131-2135, doi:10.1039/b404459b.
2. Inose, K.; Tanaka, K.; Yamada, T.; Koshino, H.; Hashimoto, M. Isolation of Peribysins O, P, and Q from Periconia macrospinosa KT3863 and Configurational Reinvestigation of Peribysin E Diacetate from Periconia byssoides OUPS-N133. *J. Nat. Prod.* **2019**, *82*, 911-918, doi:10.1021/acs.jnatprod.8b01001.
3. Athawale, P.R.; Kalmode, H.P.; Motiwala, Z.; Kulkarni, K.A.; Reddy, D.S. Overturning the Peribysin Family Natural Products Isolated from Periconia byssoides OUPS-N133: Synthesis and Stereochemical Revision of Peribysins A, B, C, F, and G. *Org Lett* **2020**, *22*, 3104-3109, doi:10.1021/acs.orglett.0c00857.
4. Che, Y.; Gloer, J.B.; Wicklow, D.T. Phomadecalins A-D and phomapentenone A: new bioactive metabolites from Phoma sp. NRRL 25697, a fungal colonist of Hypoxylon stromata. *J. Nat. Prod.* **2002**, *65*, 399-402, doi:10.1021/np010519o.
5. Nirma, C.; Eparvier, V.; Stien, D. Reactivation of antibiosis in the entomogenous fungus Chrysoporthe sp. SNB-CN74. *J. Antibiot.* **2015**, *68*, 586-590, doi:10.1038/ja.2015.36.

1D and 2D NMR Spectra of Phoenixilane A (1)

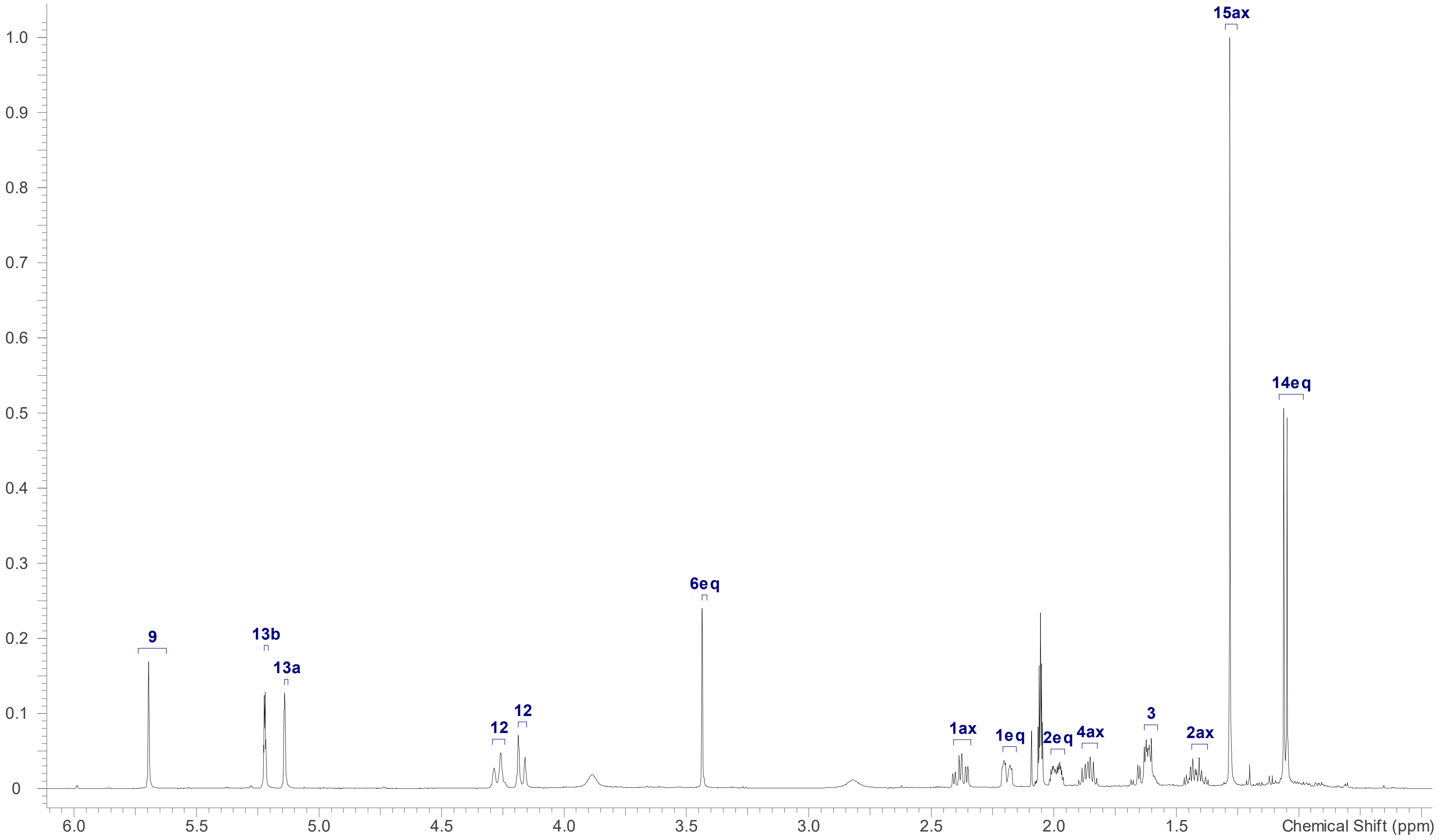

Fig. S5: ^{1}H NMR spectrum (500 MHz, acetone-d_6) of phoenixilane A (**1**).

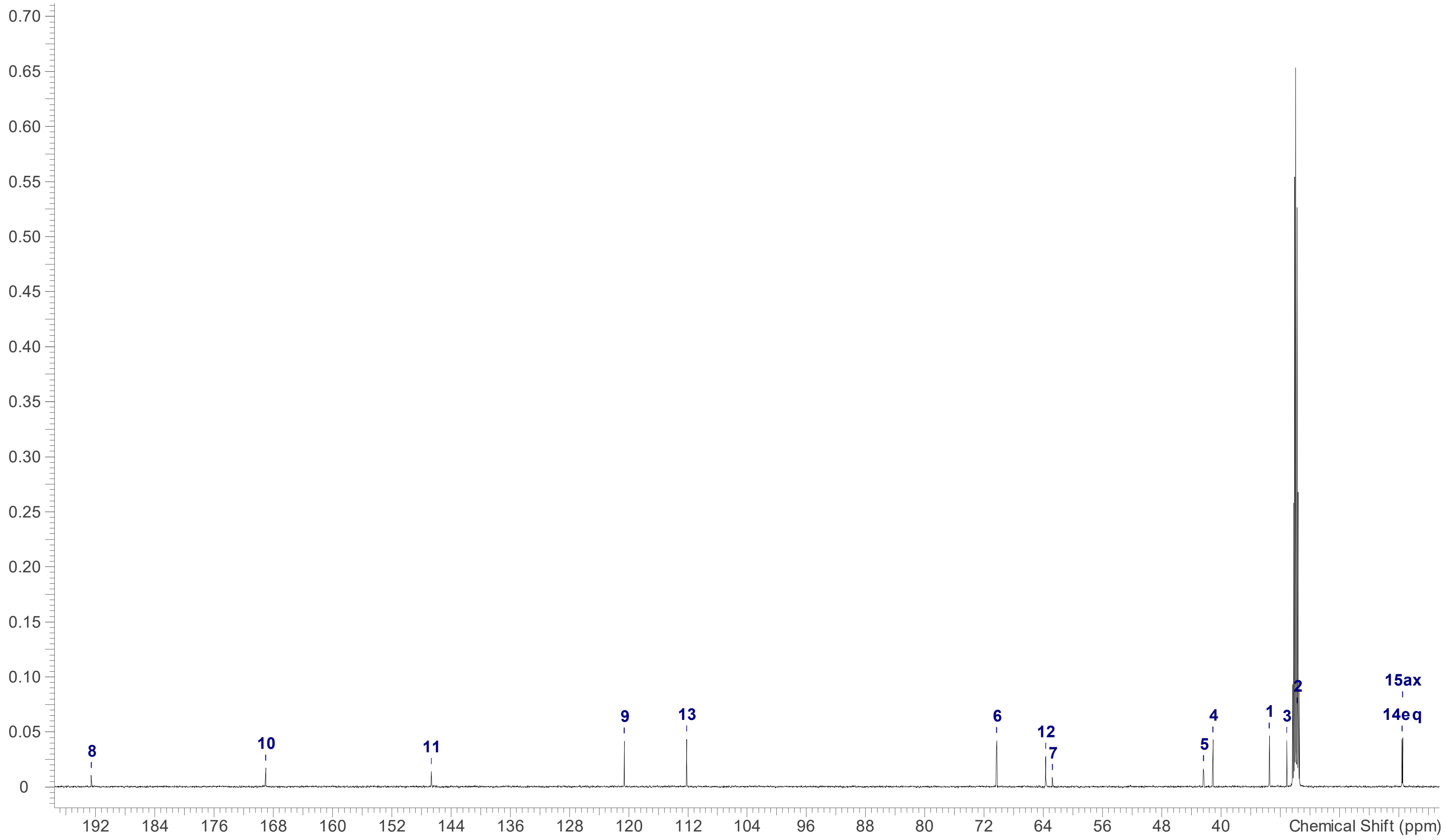

Fig. S6: ^{13}C NMR spectrum (125 MHz, acetone-d_6) of phoenixilane A (**1**).

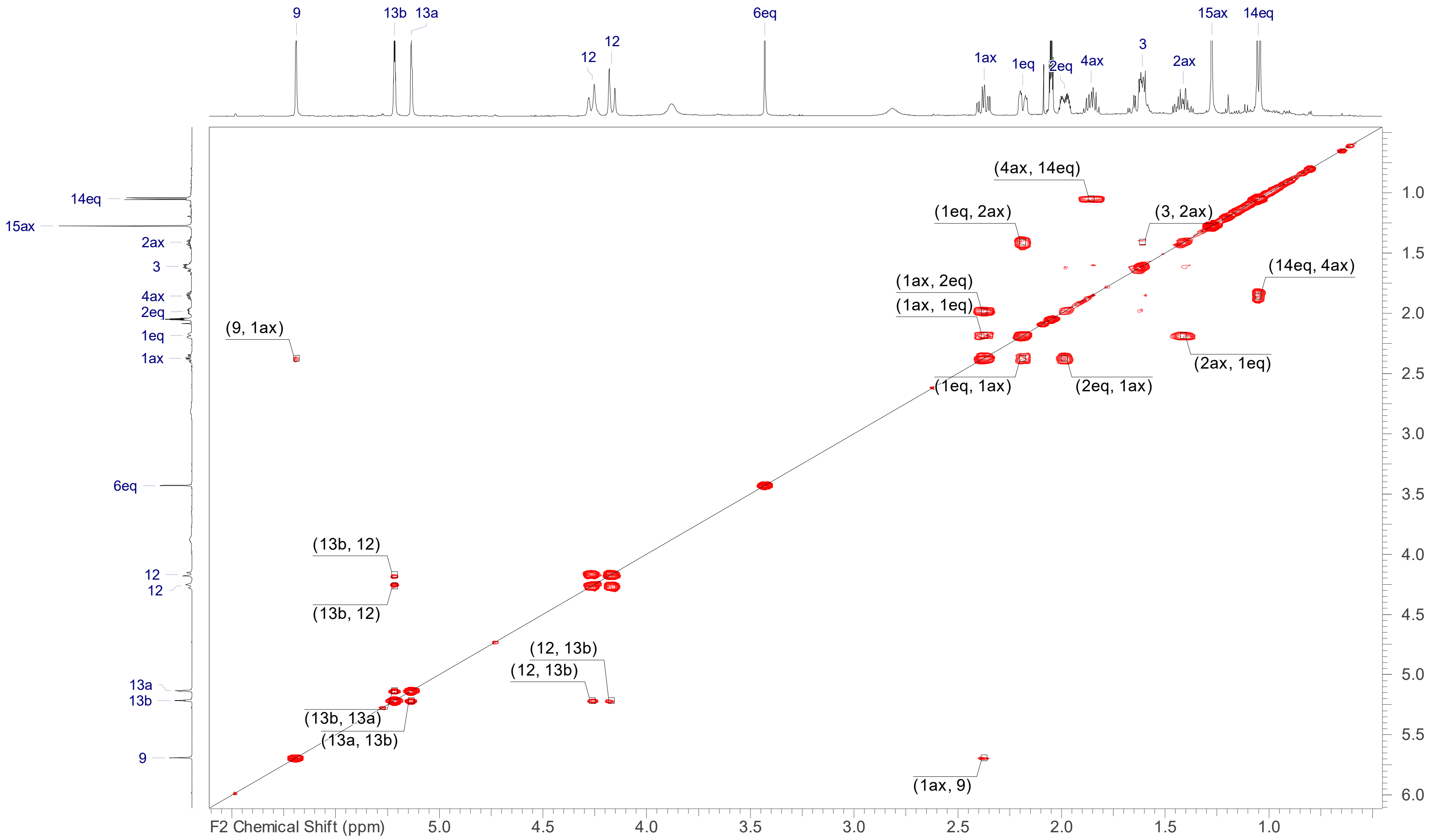

Fig. S7: $^1H/^1H$ COSY spectrum (500 MHz, acetone-d_6) of phoenixilane A (**1**).

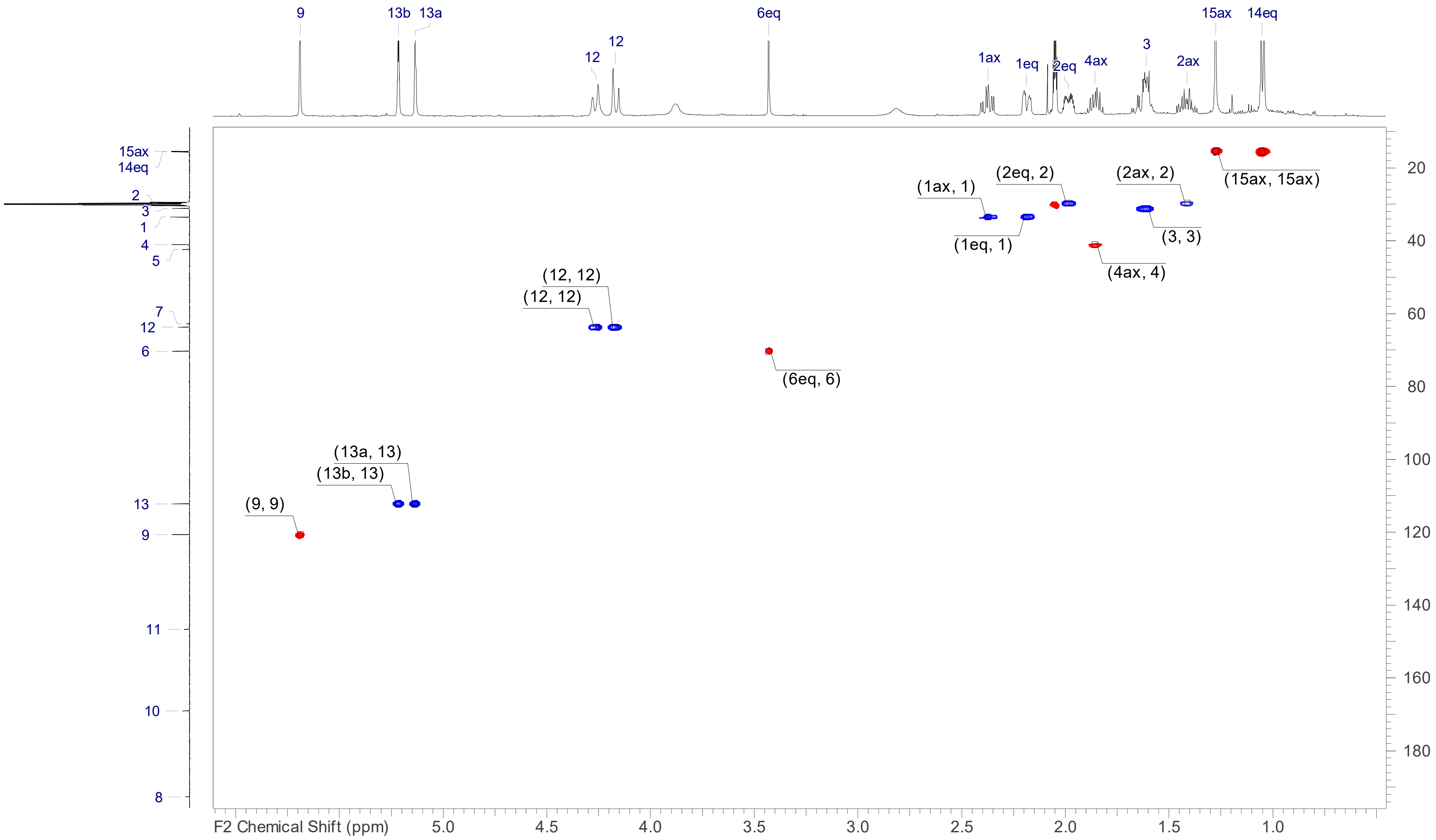

Fig. S8: $^{1}H/^{13}C$ HSQC spectrum (500 MHz, acetone-d_6) of phoenixilane A (**1**).

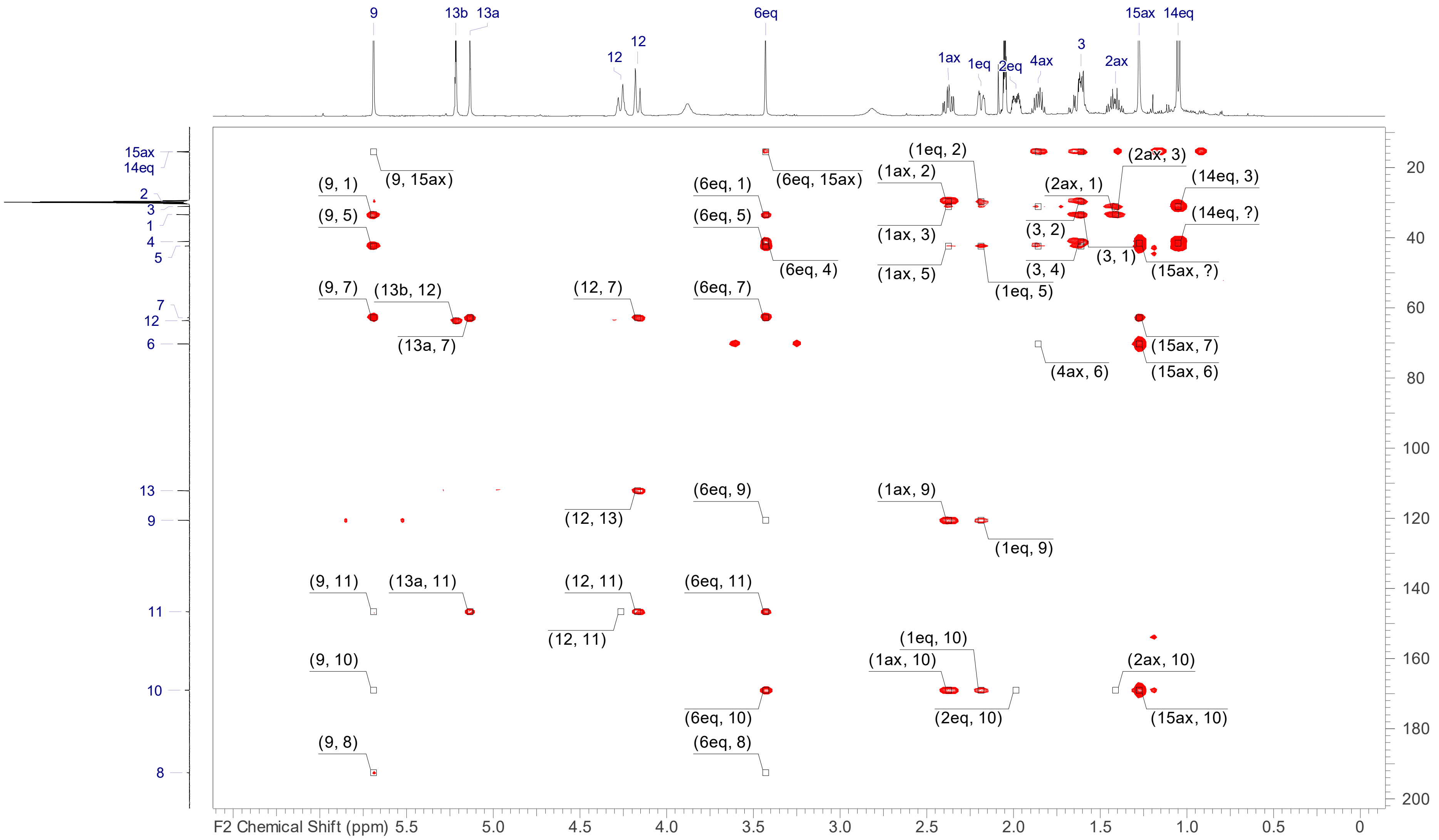

Fig. S9: ^{1}H/^{13}C HMBC spectrum (500 MHz, acetone-d_6) of phoenixilane A (**1**).

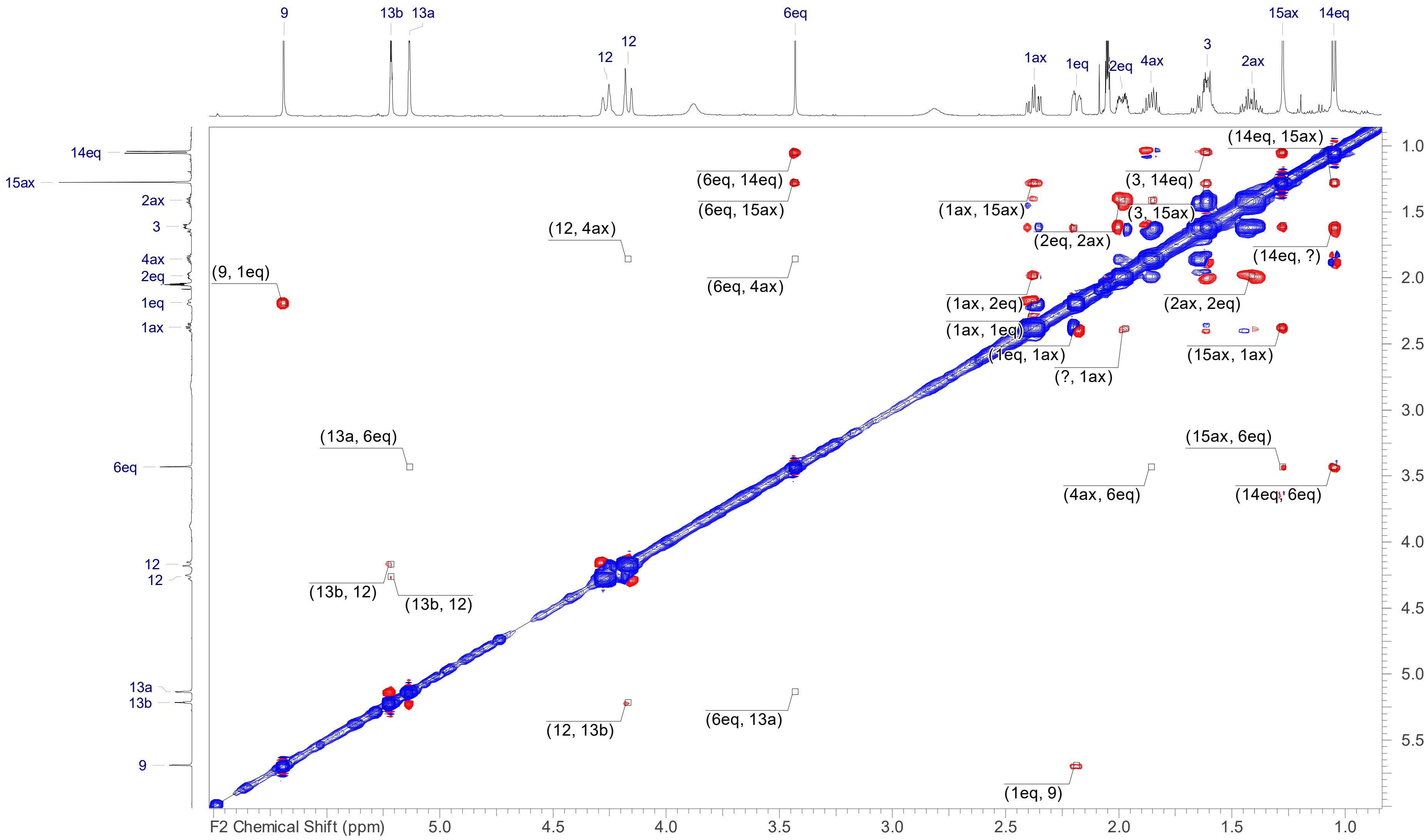

Fig. S10: $^{1}H/^{1}H$ ROESY spectrum (500 MHz, acetone-d_6) of phoenixilane A (**1**).

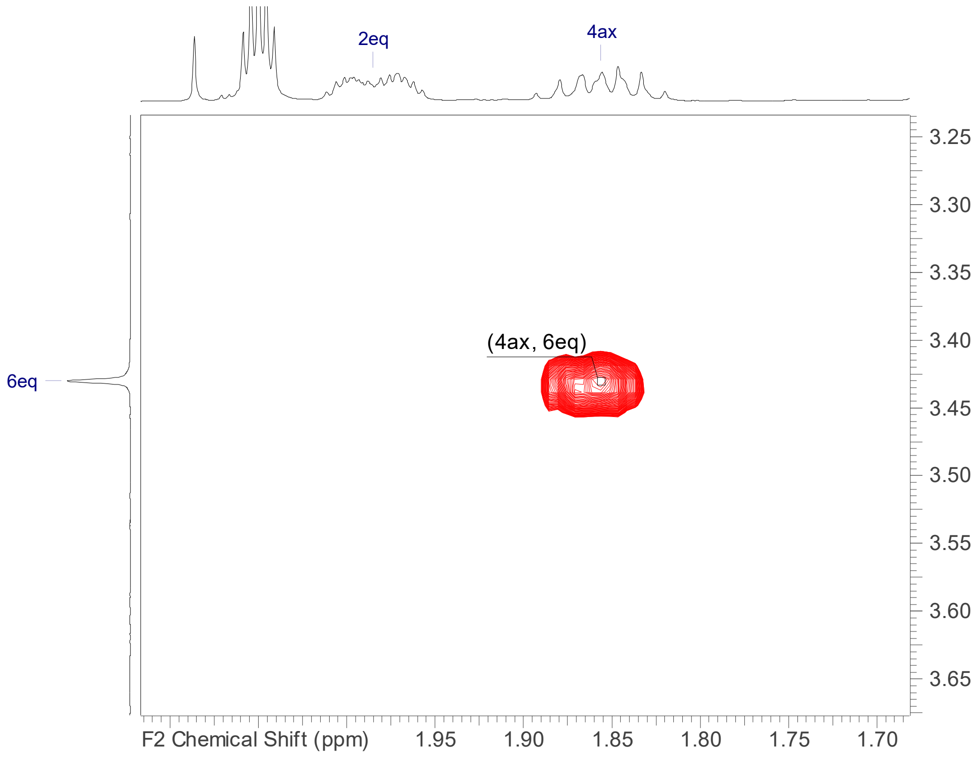

Fig. S11: Zoomed $^1H/^1H$ ROESY spectrum (500 MHz, acetone-d_6) of phoenixilane A (**1**).

1D and 2D NMR Spectra of Phoenixilane B (2)

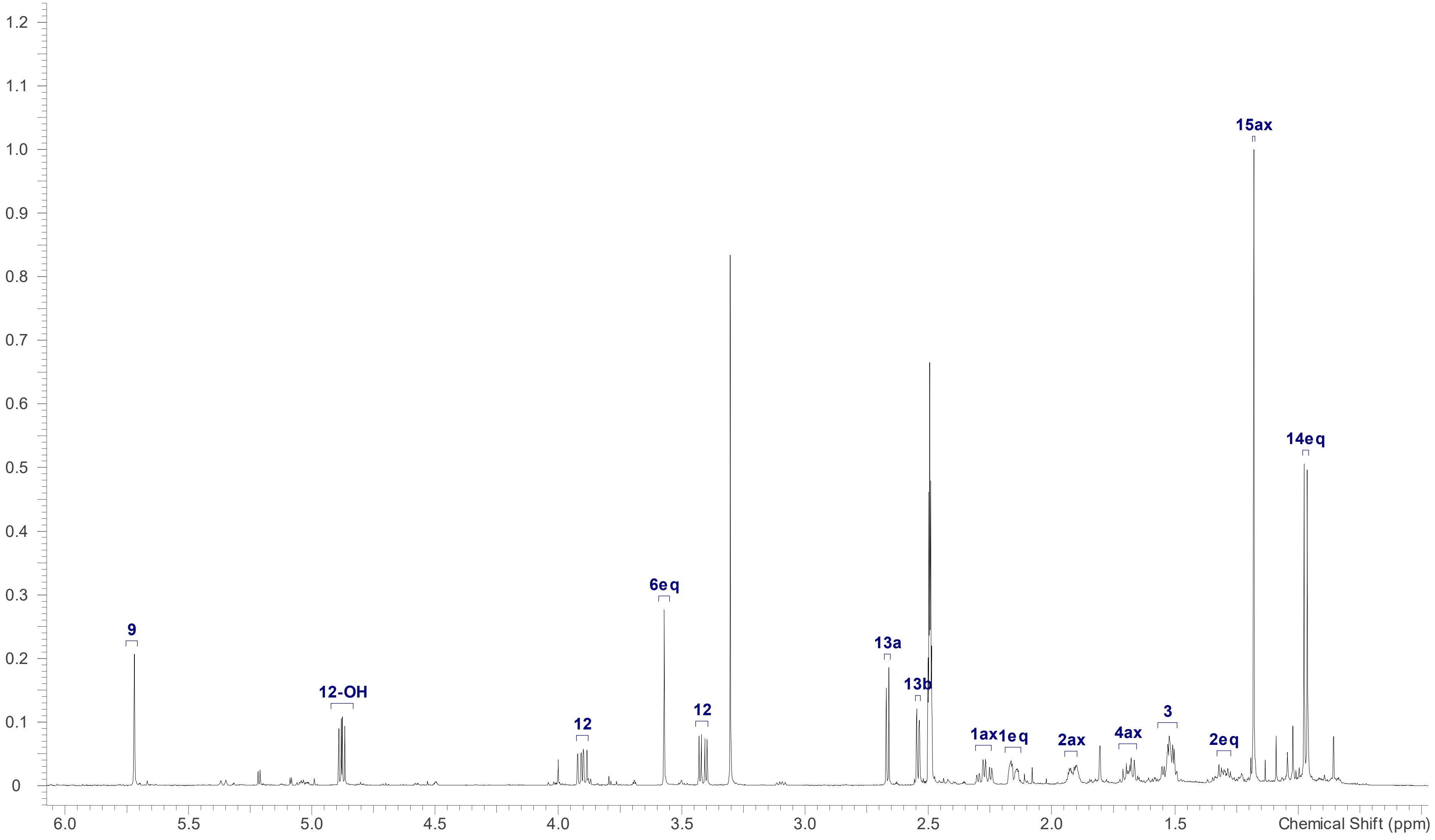

Fig. S12: 1H NMR spectrum (500 MHz, DMSO-d_6) of phoenixilane B (**2**).

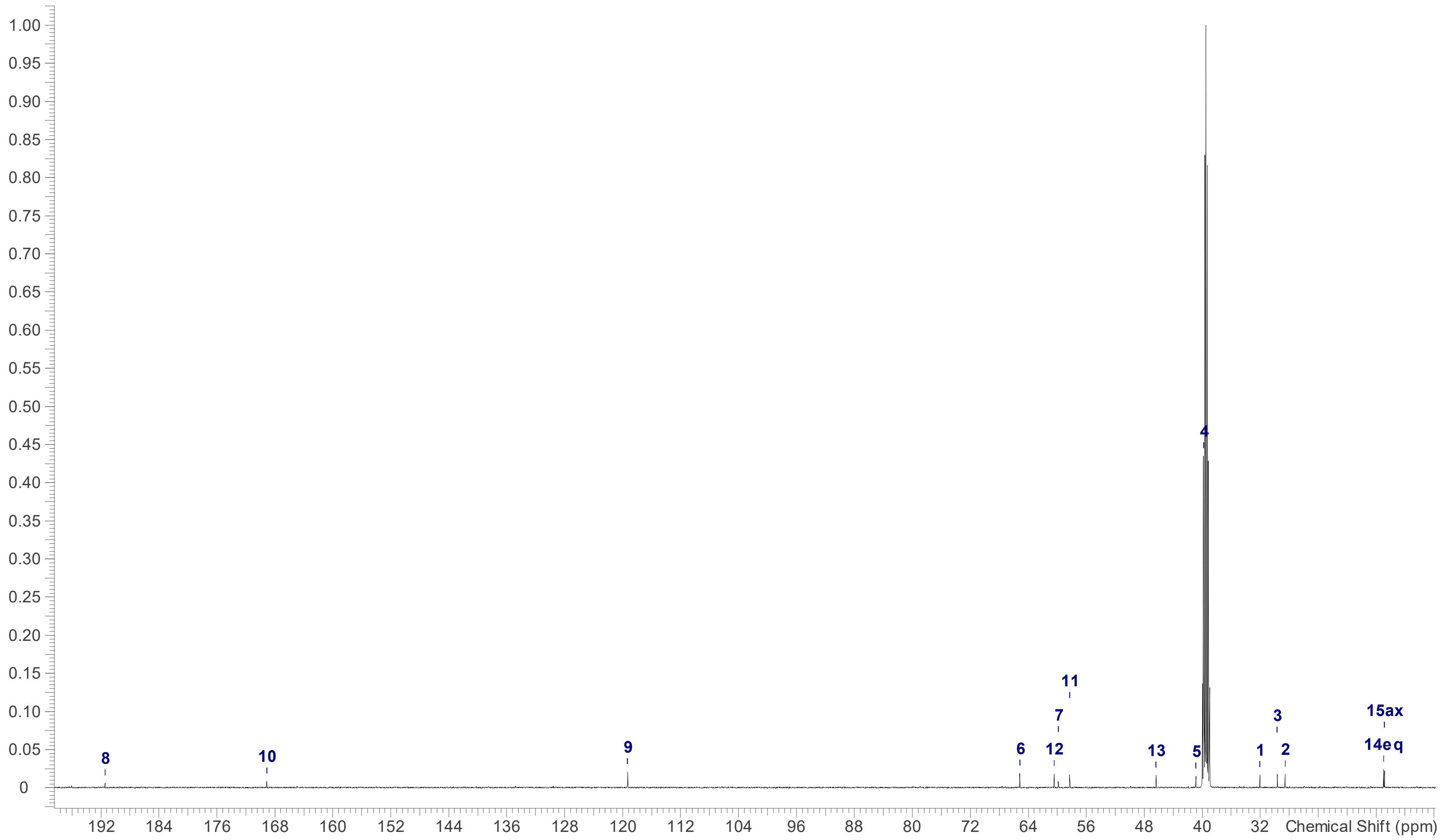

Fig. S13: ^{13}C NMR spectrum (125 MHz, DMSO-*d*$_6$) of phoenixilane B (**2**).

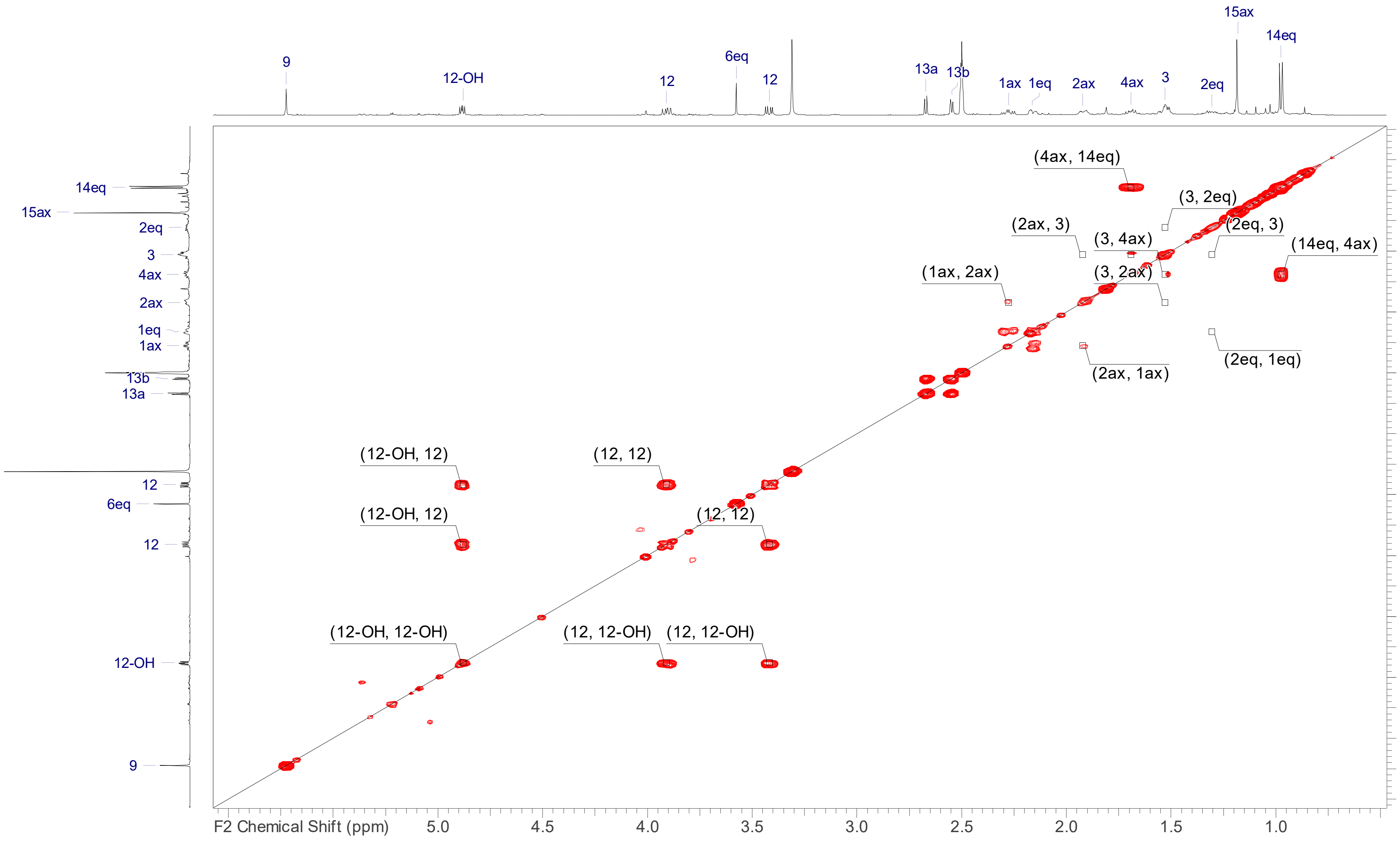

Fig. S14: $^{1}H/^{1}H$ COSY spectrum (500 MHz, DMSO-d_6) of phoenixilane B (**2**).

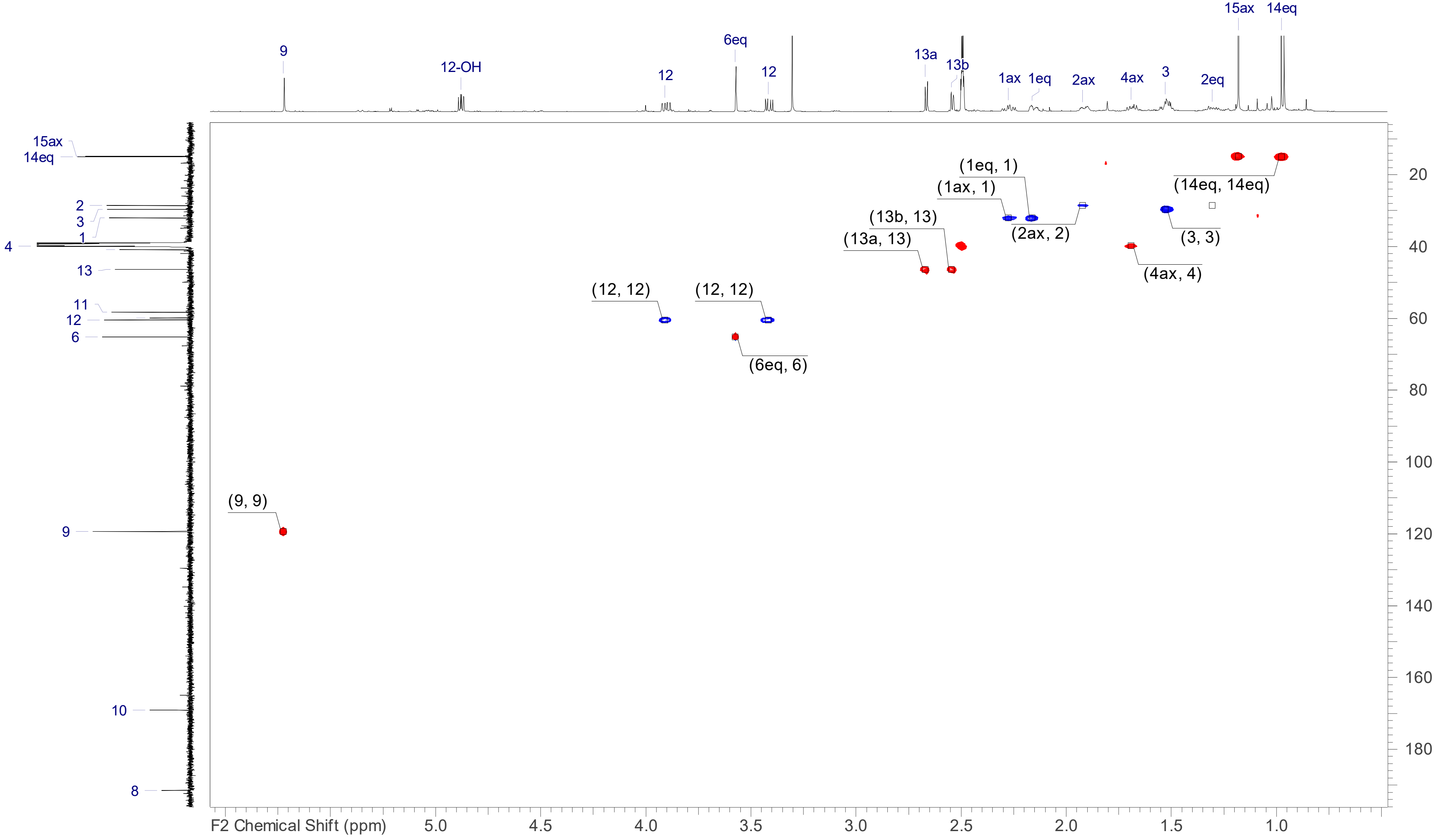

Fig. S15: $^{1}H/^{13}C$ HSQC spectrum (500 MHz, DMSO-d_6) of phoenixilane B (**2**).

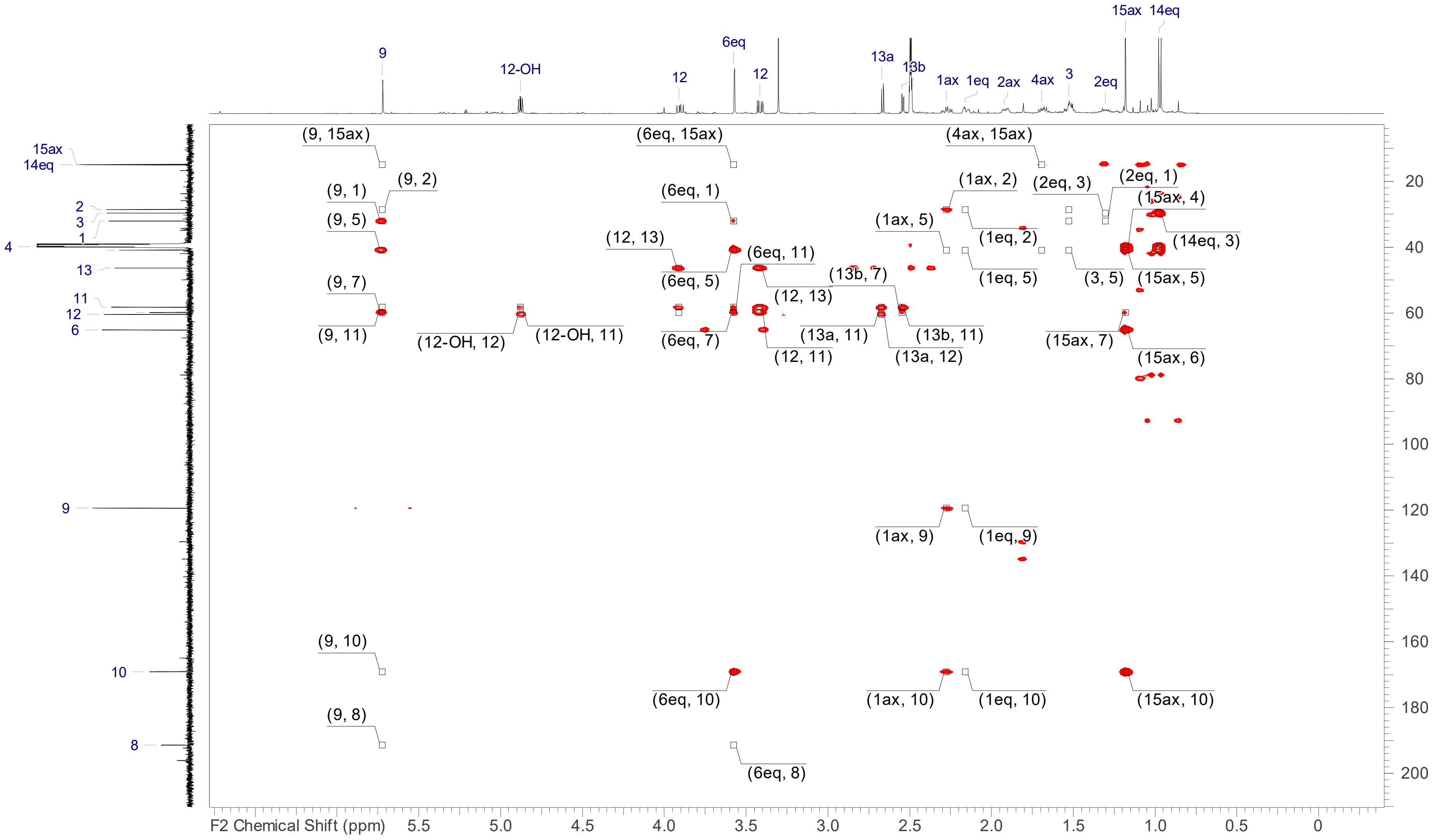

Fig. S16: $^{1}H/^{13}C$ HMBC spectrum (500 MHz, DMSO-d_6) of phoenixilane B (**2**).

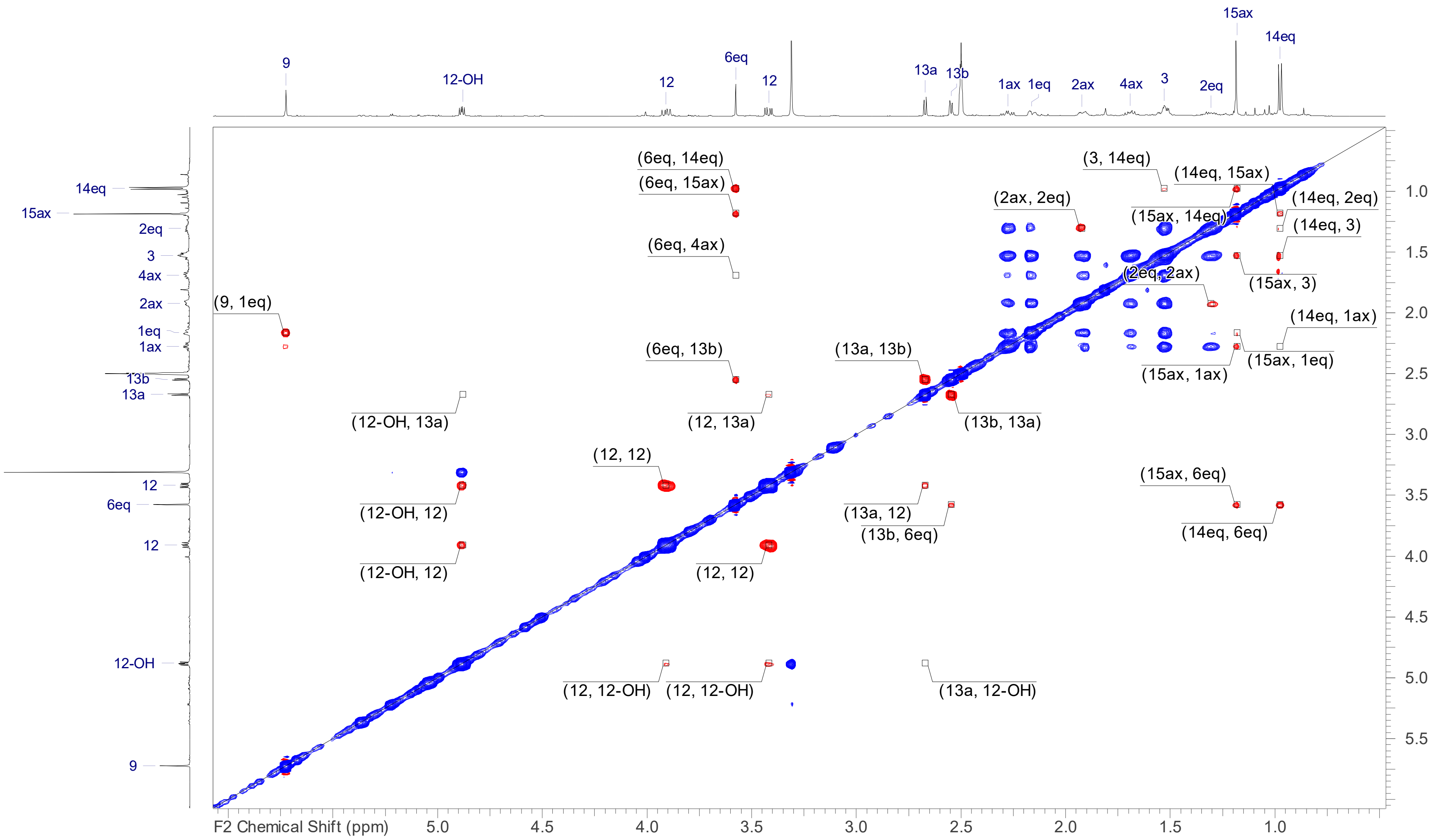

Fig. S17: $^1H/^1H$ ROESY spectrum (500 MHz, DMSO-d_6) of phoenixilane B (**2**).

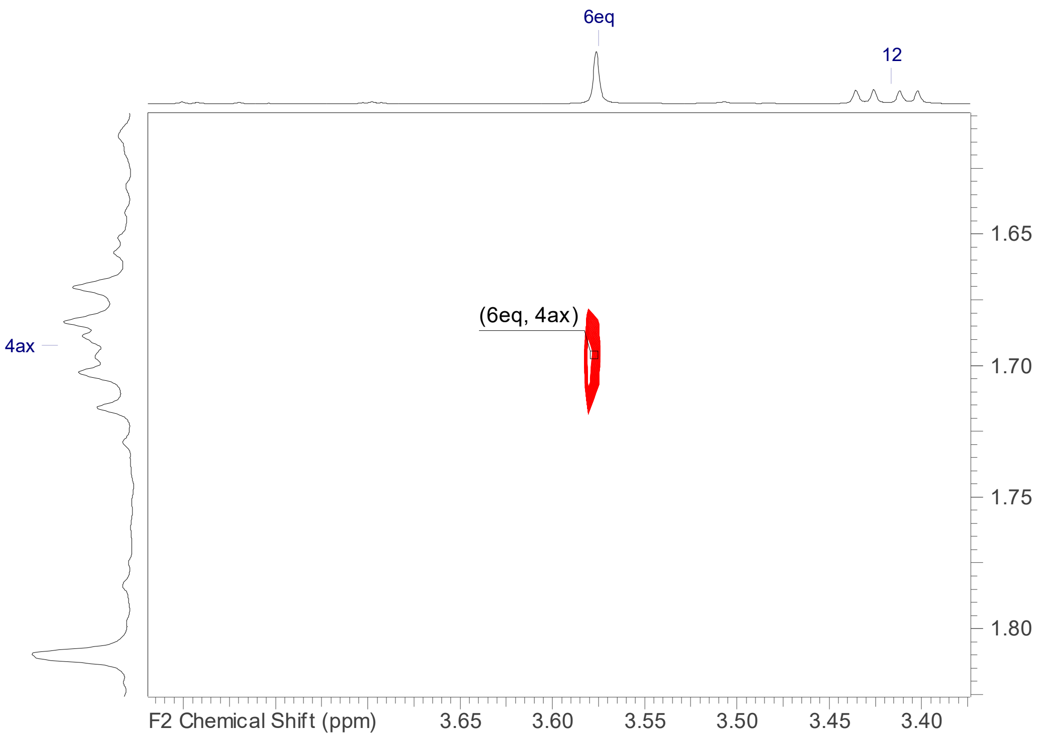

Fig. S18: Zoomed $^{1}H/^{1}H$ ROESY spectrum (500 MHz, DMSO-d_6) of phoenixilane B (**2**).

Antimicrobial and Cytotoxic Activities of Phoenixlanes A–B (1–2) and 8,9-Dehydroxylarone (4)

Table S2. Antimicrobial activities of phoenixilanes A–B (**1**–**2**) and 8,9-dehydroxylarone (**4**) as minimum inhibitory concentrations (MIC).

test organism	MIC [µg/mL]			
	**1**	**2**	**4**	**reference**
Bacillus subtilis	> 66.7	> 66.7	> 66.7	8.3 [1]
Staphylococcus aureus	> 66.7	> 66.7	> 66.7	0.8 [1]
Micrococcus luteus	> 66.7	> 66.7	> 66.7	0.8 [1]
Chromobacterium violaceum	> 66.7	> 66.7	> 66.7	0.8 [1]
Escherichia coli	> 66.7	> 66.7	> 66.7	6.7 [1]
Pseudomonas aeruginosa	> 66.7	> 66.7	> 66.7	0.4 [2]
Mycolicibacterium smegmatis	> 66.7	> 66.7	> 66.7	1.7 [3]
Candida albicans	> 66.7	> 66.7	> 66.7	66.7 [4]
Schizosaccharomyces pombe	> 66.7	> 66.7	> 66.7	8.3 [4]
Mucor hiemalis	> 66.7	> 66.7	66.7	33.3 [4]
Pichia anomala	> 66.7	> 66.7	> 66.7	16.7 [4]
Rhodotorula glutinis	> 66.7	> 66.7	> 66.7	8.3 [4]

[1] oxytetracycline, [2] gentamicin, [3] kanamycin, [4] nystatin

Table S3. Cytotoxicities of phoenixilanes A–B (**1**–**2**) and 8,9-dehydroxylarone (**4**) as half maximal inhibitory concentrations (IC_{50}). n.i.: no inhibition observed. n.d.: not determined

cell line		Cytotoxicity (IC_{50}) [µM]			
		**1**	**2**	**4**	**reference** [1]
L929	mouse fibroblasts	n.i.	31.1	n.i.	0.00004
KB 3.1	human endocervical adenocarcinoma (AC)	n.i.	68.2	n.i.	0.00063
PC-3	human prostate AC	n.d.	45.5	n.d.	0.00028
SK-OV-3	human ovary AC	n.d.	68.2	n.d.	0.00024
MCF-7	human breast AC	n.d.	14.4	n.d.	0.00005
A431	human squamous AC	n.d.	17.4	n.d.	0.00005
A549	human lung carcinoma	n.d.	31.4	n.d.	0.00008

[1] epothilon B

MUSCLE Alignments of ITS, LSU, RPB2, TUB2 gene regions from *Stromatoneurospora phoenix*

MUSCLE alignments of the four gene loci ITS, LSU, RPB2 and TUB2 of the conducted molecular phylogenetic analysis are attached separately as `.fasta` files.
